# Mpox Resurgence: A Multifaceted Analysis for Global Preparedness

**DOI:** 10.3390/v16111737

**Published:** 2024-11-05

**Authors:** Fatouma Mohamed Abdoul-Latif, Ayoub Ainane, Houda Mohamed, Ali Merito Ali, Ibrahim Houmed Aboubaker, Pannaga Pavan Jutur, Tarik Ainane

**Affiliations:** 1 Medicinal Research Institute, Center for Research and Study of Djibouti, Djibouti P.O. Box 486, Djibouti; abdoulhouda@yahoo.fr (H.M.); alimerito@hotmail.fr (A.M.A.); ibrahimhoumed@yahoo.fr (I.H.A.); 2 Superior School of Technology, University of Sultan Moulay Slimane, P.O. Box 170, Khenifra 54000, Morocco; a.ainane@usms.ma; 3 Peltier Hospital of Djibouti, Djibouti P.O. Box 2123, Djibouti; 4 Omics of Algae Group, Industrial Biotechnology, International Centre for Genetic Engineering and Bio-Technology, Aruna Asaf Ali Marg, New Delhi 110067, India; pavan.jutur@icgeb.org

**Keywords:** mpox (MPXV), epidemiology, diagnostics, antiviral therapy, vaccination, public health

## Abstract

This study provides an in-depth analysis of mpox, encompassing its history, characteristics, epidemiology, diagnostics, treatment options, and the ongoing evolution of the virus and its transmission dynamics. Mpox, though once successfully eradicated, has re-emerged with new modes of transmission and a broader host range. Genomic analyses have revealed the virus’s adaptability, posing challenges for diagnostics and vaccine efficacy. The epidemiology has shifted from sporadic zoonotic transmission in rural Africa to a significant presence in urban areas, particularly impacting high-risk populations. Advancements in diagnostics and therapeutics offer hope, but challenges persist. This work underscores the critical need for enhanced surveillance, vaccination strategies, and continued research to bolster global health systems and preparedness for future outbreaks.

## 1. Introduction

Mpox, a zoonotic orthopoxvirus closely related to the eradicated smallpox virus, has recently garnered global attention due to its resurgence and unprecedented spread beyond its endemic regions [1]. Originally confined to Central and West Africa, where it caused isolated outbreaks primarily through zoonotic transmission, the virus has now demonstrated its capacity for sustained human-to-human transmission, making it a pressing global health concern [2].

This epidemiological shift, combined with the virus’s potential for genetic adaptation and the waning immunity in populations no longer vaccinated against smallpox, underscores the need for an in-depth understanding of mpox. This review aims to address key research questions by analyzing the virus’s molecular properties, transmission dynamics, and clinical characteristics, while also evaluating prevention and control strategies adapted to its evolving nature. Such a comprehensive approach is important in identifying critical knowledge gaps and guiding the development of effective public health interventions.

**Figure 1:**
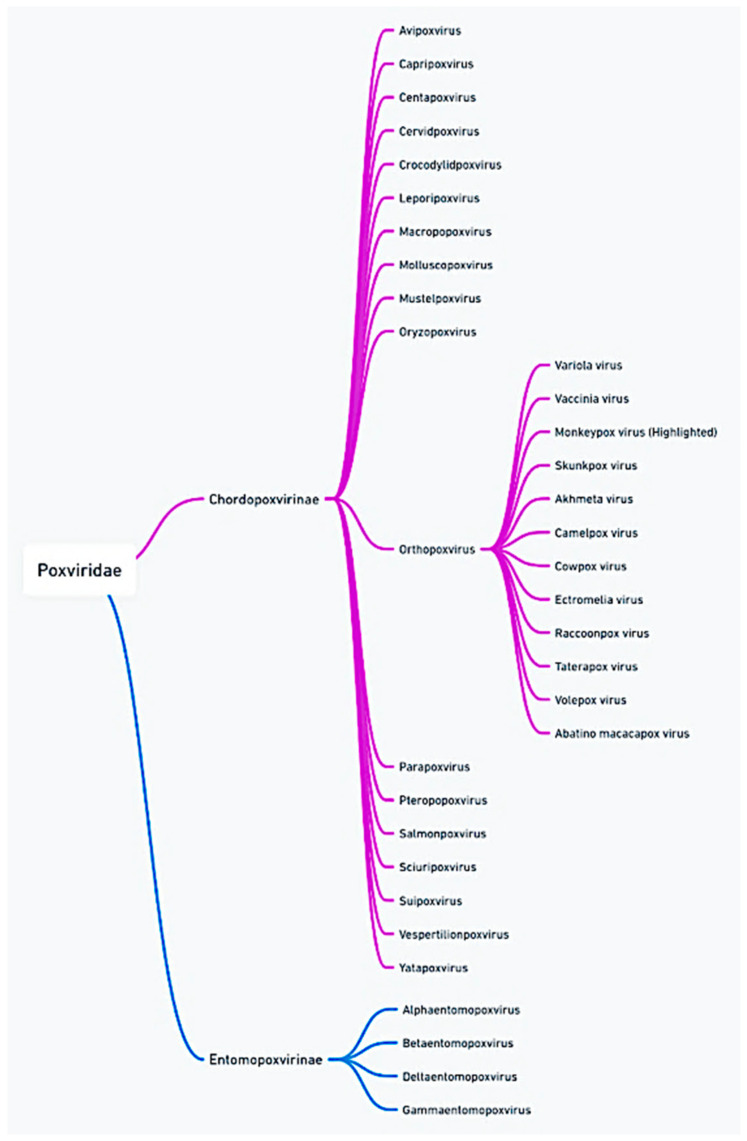
The taxonomic classification of MPXV showcases its position within the Poxviridae family and its relationship to other notable viruses like the variola (smallpox) and vaccinia viruses. This emphasizes the evolutionary connections and shared characteristics among these viruses.

The re-emergence of mpox poses unique challenges that require rethinking current public health strategies, particularly in the context of changing transmission patterns and a potentially broader host range. Additionally, the possibility of genetic mutations affecting viral virulence, transmissibility, and vaccine efficacy calls for vigilant surveillance and continued research [3]. Addressing these challenges will necessitate a coordinated, multidisciplinary approach involving virology, epidemiology, clinical medicine, and public health to better anticipate and mitigate future outbreaks, ultimately safeguarding the global population [4] (Figure 1; Supplementary Figure S1).

## 2. Molecular Characteristics of Mpox

### 2.1. Genomic Structure

**Figure 2:**
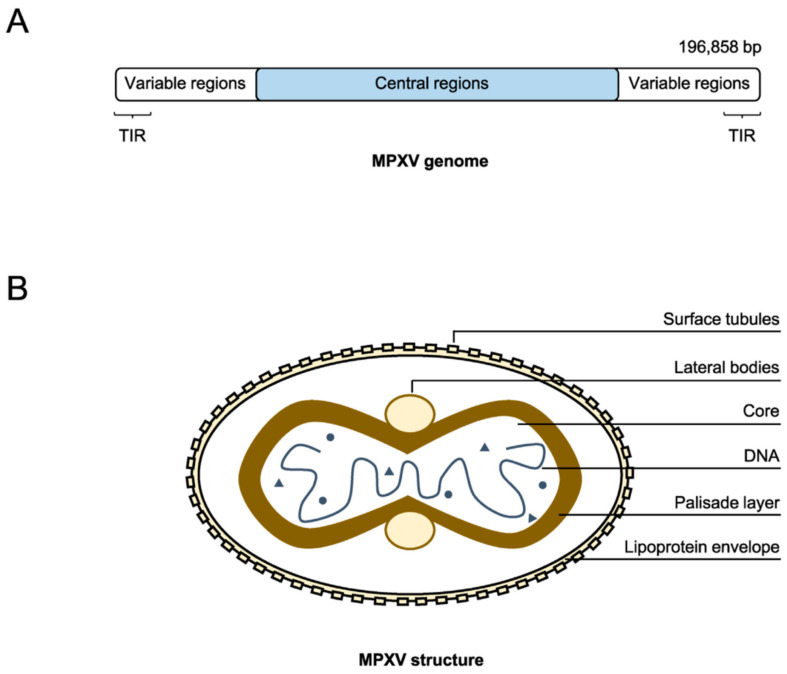
Structure and genomic organization of MPXV. (**A**) The MPXV genome features a large, conserved central region encoding essential factors flanked by two variable zones with inverted terminal repeats (ITRs). (**B**) The MPXV virion has five main components: a double-concave dumbbell-shaped core, a lipoprotein membrane, lateral bodies, surface tubules, and its genomic DNA. (Figure from Ref. [9]).

The mpox virus (MPXV) possesses a large, double-stranded DNA genome that is approximately 197 kilobases (kb) in length [5]. This places it among the largest known viral genomes. The genome exhibits a well-defined structure, comprising a central conserved core region flanked by two variable terminal regions containing inverted terminal repeats (ITRs) (Figure 2) [6]. The core region encodes essential enzymes and structural proteins vital for viral replication, transcription, and virion assembly [6]. In contrast, the less conserved terminal regions harbor genes associated with pathogenicity and host range, including those involved in immune evasion and virulence [7]. These terminal regions notably contain genes that encode proteins capable of inhibiting host cytokines like interleukin-1β (IL-1β) and interferons, critical players in the antiviral immune response [8].

MPXV shares a high degree of sequence similarity with other *Orthopoxviruses*, particularly the variola (smallpox) and vaccinia viruses. The central region of the MPXV genome exhibits approximately 96.3% nucleotide sequence identity with the variola virus, underscoring their close evolutionary relationship. However, the terminal regions, enriched in virulence and host specificity genes, display more significant divergence [10]. Specific genes in MPXV, such as those encoding certain immune-modulatory proteins, are absent or fragmented in the variola virus genome, suggesting differences in their pathogenic mechanisms [10].

Several open reading frames (ORFs) within the MPXV genome are essential for its replication and pathogenicity. The gene encoding the VP37 protein is particularly noteworthy due to its role in viral envelope formation, which is essential for virion stability and infectivity [11]. Other significant ORFs encode proteins involved in DNA repair, replication fidelity, and modulation of host cell processes, collectively contributing to the virus’s ability to persist in diverse hosts and environments.

The genetic similarities between MPXV and other *Orthopoxviruses*, like the vaccinia virus, extend to their immune evasion mechanisms. Both viruses possess genes encoding proteins that bind and inhibit host cytokines, dampening the immune response and facilitating viral replication [12]. This genetic arsenal highlights *Orthopoxviruses*’ adaptability and capacity to cause persistent infections across a broad host range.

### 2.2. Evolutionary Clades and Mutations

The mpox virus (MPXV) is divided into two distinct evolutionary clades: Clade I, also known as the Congo Basin clade, and Clade II, or the West African clade. These clades differ in geographic and genetic characteristics, which influence their epidemiological and clinical features [13]. Clade I, historically associated with the Congo Basin, is known for its increased virulence and higher mortality rate. This is primarily due to its ability to cause severe disease, with a higher risk of complications such as encephalitis and secondary bacterial infections [13]. In contrast, Clade II, which is more prevalent in West Africa, tends to cause milder forms of the disease with a lower fatality rate [13]. Its transmission is often limited to sporadic outbreaks in rural areas, with minimal human-to-human transmission [13]. Genetically, the two clades exhibit notable differences in their nucleotide sequences, particularly in regions of the genome associated with virulence and host interaction [14]. Comparative studies have shown that Clade I and Clade II differ by approximately 0.55% in their nucleotide sequences, with Clade I possessing additional gene duplications and mutations [14]. These genetic differences, particularly in the genes coding for immune-modulating proteins such as those that inhibit interferon and interleukin signaling, play a key role in evading the host’s antiviral defenses [14]. Clade I appears to be better equipped to evade the immune response, which explains its higher virulence compared to Clade II [14]. Recent outbreaks in 2022–2023 have highlighted the emergence of new mutations within the MPXV genome, particularly within Clade II, which was at the center of these outbreaks [15,16]. These mutations primarily affect genes involved in viral entry and replication, such as those encoding surface glycoproteins and enzymes responsible for DNA replication [17]. These genetic alterations raise concerns about the potential for increased viral transmissibility and changes in virulence [17]. For example, certain mutations in viral glycoproteins have been associated with enhanced binding affinity to human cell receptors, facilitating more efficient viral entry and replication in human hosts [17]. Furthermore, mutations in the viral DNA polymerase gene may improve replication efficiency, resulting in higher viral loads and increased viral shedding [18]. This could lead to more frequent human-to-human transmission, especially in densely populated areas or among immunocompromised individuals [18]. Regarding congenital mpox syndrome, this is a rare but serious complication that occurs when the virus is transmitted from mother to fetus during pregnancy. A notable case was documented in the Democratic Republic of the Congo in 2008, where a 21-week stillborn fetus was infected, likely by Clade I, which was endemic in the region at the time [15]. The fetus exhibited typical signs of infection, including widespread skin lesions and hydrops, an abnormal accumulation of fluid in the body [16]. Postmortem analyses confirmed the presence of the virus in the fetal tissues, particularly in the skin, liver, and placenta [15]. The placenta showed signs of infection, with visible hemorrhages on the maternal surface and a significant immune response from the placental cells [15]. This intrauterine infection led to severe outcomes, including spontaneous abortions and stillbirths, particularly associated with Clade I, which is known for its high perinatal mortality rate [15]. Although congenital mpox syndrome is rare, it poses a serious threat to pregnant women, especially in areas with limited medical resources. It is good for healthcare professionals to be aware of this risk, particularly during outbreaks, to better protect mothers and their infants from the severe complications this infection can cause [15] (Table 1; Supplementary Figure S2).

### 2.3. Mechanisms of Viral Infection

**Figure 3:**
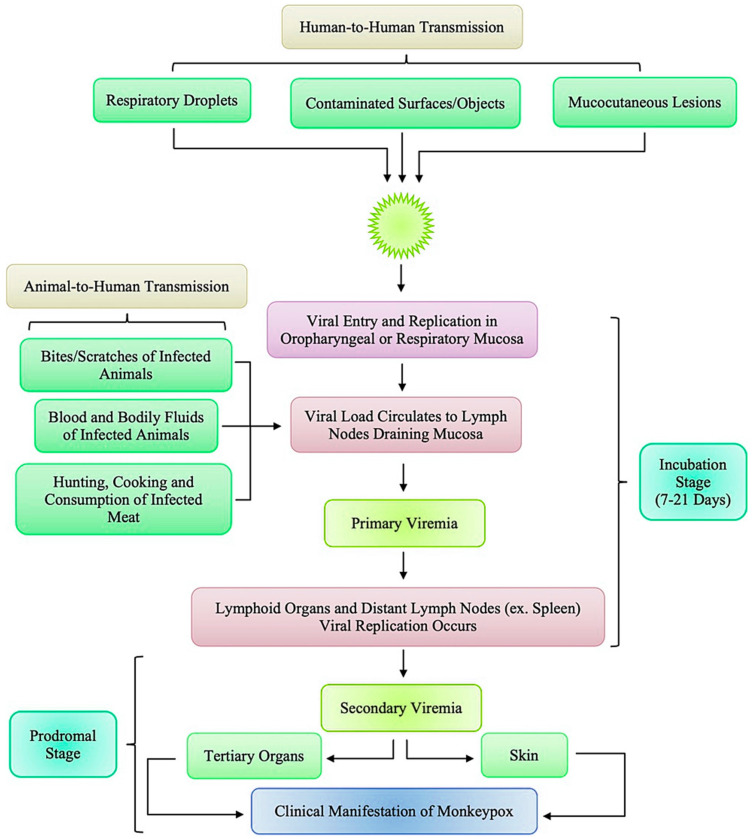
Stages involved in Mpox pathogenesis, from viral entry and replication to clinical manifestations and potential complications. The diagram highlights the interplay between the virus and the host immune response, showcasing viral immune evasion and tissue damage. From Ref. [20].

The pathogenesis of the mpox virus (MPXV) involves a complex interaction between the virus and host cells, beginning with the virus’s entry into the host and culminating in viral replication and spread. MPXV, like other *Orthopoxviruses*, primarily enters host cells through endocytosis, a process mediated by the interaction between viral surface proteins and specific receptors on the host cell membrane [16]. These surface proteins include viral glycoproteins such as A33, B5, and L1, which play essential roles in attachment and entry. Upon binding to the host cell, the virus is internalized through a clathrin-mediated endocytic pathway, leading to the uncoating of the viral envelope and the viral core’s release into the cytoplasm [16] (Figure 3).

Once inside the host cell, MPXV begins its replication cycle in the cytoplasm, a unique feature of poxviruses, which do not require the host nucleus for replication [19]. The viral core contains the necessary transcription machinery, including viral RNA polymerase, to initiate early gene transcription immediately after entry. Early viral genes encode proteins essential for DNA replication, immune evasion, and intermediate and late gene product production. DNA replication occurs within cytoplasmic factories formed by viral and host cell components. The newly synthesized viral DNA is packaged into viral particles assembled in the cytoplasm and released from the host cell to infect neighboring cells [19].

**Figure 4:**
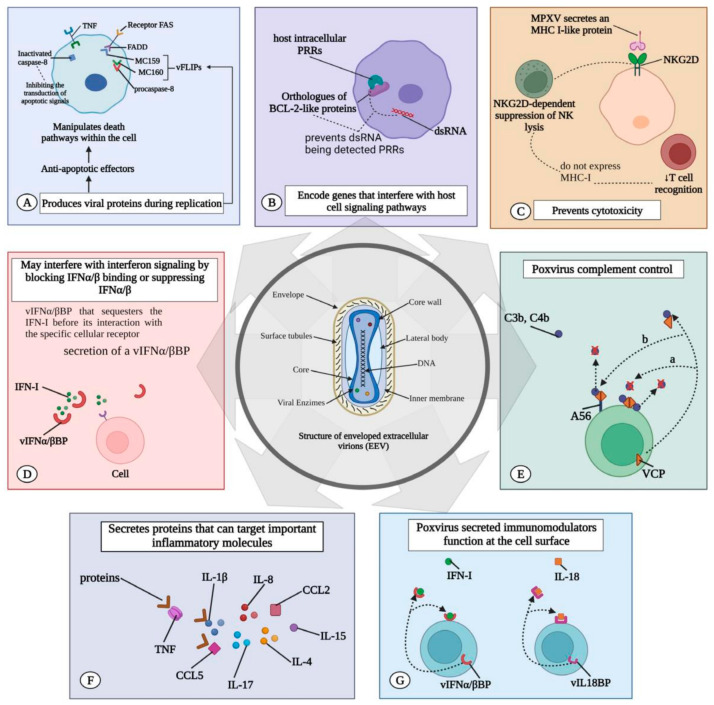
Visual representation of MPXV strategies to evade the host immune response, highlighting viral proteins inhibiting key immune signaling pathways like cytokine neutralization and interferon/complement inhibition. The diagram illustrates how these mechanisms contribute to establishing a productive infection and causing disease. (**A**) The virus enters the host cell; (**B**) the virus uncoats and releases its core into the cytoplasm; (**C**) the virus begins replicating its DNA and producing viral proteins; (**D**) some viral proteins are secreted from the infected cell; (**E**) secreted viral proteins bind to and inhibit host cytokines, disrupting immune signaling; (**F**) other viral proteins remain within the infected cell; and (**G**) intracellular viral proteins inhibit host cell processes, such as apoptosis and interferon responses, further evading the immune system. (Figure from Ref. [22]).

The ability of MPXV to evade the host immune response is a critical factor in its pathogenesis and virulence. The virus has evolved multiple strategies to inhibit host immune defenses, primarily through the action of viral proteins that interfere with critical immune signaling pathways [19]. One such approach involves the production of viral proteins that mimic host cytokine receptors, such as the viral TNF receptor (CrmB) and the IL-1β receptor (B15R). These viral proteins bind to and neutralize cytokines like TNF-α and IL-1β, which are important for initiating an adequate inflammatory response. MPXV effectively dampens the host’s ability to mount an immune response by inhibiting these cytokines, allowing the virus to replicate more efficiently and spread within the host [21] (Figure 4).

Additionally, MPXV produces proteins that directly inhibit the activation of host immune cells [21]. The viral protein C3L, for example, inhibits the complement system, a critical component of the innate immune response that helps clear pathogens through opsonization and lysis. MPXV reduces the effectiveness of the host’s immune clearance mechanisms by inhibiting complement activation. Another significant virulence factor is the viral E3L protein, which inhibits the host’s interferon response. E3L binds to double-stranded RNA (dsRNA) produced during viral replication, preventing the activation of interferon-stimulated genes (ISGs) that would otherwise enhance the host’s antiviral defenses. This inhibition of the interferon response facilitates viral replication and contributes to the virus’s systemic spread within the host [21].

The combined effects of these immune evasion strategies enable MPXV to establish a productive infection in the host, leading to the characteristic clinical manifestations of mpox. The ability of the virus to modulate host immune responses through multiple pathways underscores its virulence. It highlights the challenges in developing effective treatments that can enhance host immunity without exacerbating the disease. Understanding these mechanisms at a molecular level is important for developing targeted antiviral therapies that can disrupt these viral processes and mitigate the severity of mpox infections [20].

### 2.4. Environmental Stability and Resistance

The mpox virus (MPXV) exhibits significant environmental stability due to its persistence and transmission in both endemic and non-endemic regions [22]. Like other *Orthopoxviruses*, MPXV possesses a resilient structure that enables it to survive outside the host for extended periods [22]. This stability is primarily attributed to the virus’s complex and robust outer envelope, protecting it against environmental stressors such as temperature fluctuations, desiccation, and chemical disinfectants [22]. Studies have demonstrated that MPXV can remain viable on surfaces like bedding, clothing, and other fomites for several weeks under favorable conditions, particularly in more relaxed, drier environments where viral degradation is minimized [22]. This characteristic underscores the importance of rigorous sanitation and disinfection protocols to prevent the spread of the virus in both healthcare settings and the community at large.

The virus’s resistance to disinfectants further complicates efforts to control its spread [23]. MPXV shows varying degrees of resistance to different classes of disinfectants [23]. While susceptible to high-level disinfectants containing chlorine, ethanol, and hydrogen peroxide, it is more resistant to lower-level disinfectants, particularly those not applied correctly or in a sufficient concentration [23]. For example, quaternary ammonium compounds, commonly used in many disinfectants, may only be fully effective against MPXV if used under strict guidelines [23]. This resistance necessitates carefully designed and implemented disinfection protocols to ensure complete viral inactivation, particularly in high-contamination-risk environments.

Environmental conditions, including temperature, humidity, and ultraviolet (UV) light exposure, significantly influence MPXV’s stability and persistence outside the host [24]. MPXV is more stable at lower temperatures, which helps explain its persistence in cooler climates [24]. For instance, the virus can remain infectious for several months when stored at 4 °C to 10 °C [24]. In contrast, higher temperatures accelerate viral degradation, though complete inactivation typically requires prolonged exposure to temperatures above 60 °C [24]. Humidity also affects viral stability; lower humidity levels tend to preserve infectivity, whereas high moisture, especially with heat, can lead to faster inactivation [24].

Ultraviolet (UV) light, particularly UV-C, is highly effective at inactivating MPXV [25]. UV-C light directly damages viral DNA, forming thymine dimers that inhibit viral replication [25]. However, UV light’s effectiveness depends on the exposure intensity and duration, as well as the presence of protective barriers shielding the virus [25]. For example, MPXV particles shielded by organic material or embedded in dust may be less susceptible to UV inactivation.

The combination of environmental stability, resistance to certain disinfectants, and the influence of environmental factors like temperature and UV light underscores the importance of maintaining stringent ecological controls to prevent MPXV spread [26]. This includes using effective disinfectants, proper sanitation practices, and environmental monitoring to reduce the transmission risk in healthcare and community settings [26]. Understanding the factors contributing to MPXV’s environmental resilience is critical for developing effective public health strategies to control and prevent future outbreaks.

## 3. Epidemiology of Mpox

### 3.1. Historical Epidemiology (1970–2020)

Mpox, a zoonotic disease primarily affecting Central and West Africa, was first identified in humans in 1970 in the Democratic Republic of the Congo (DRC). In the early years after its discovery, mpox outbreaks were sporadic and mainly limited to remote, forested regions where human populations had close contact with potential animal reservoirs, such as rodents and non-human primates. During the 1970s and 1980s, most reported cases occurred in rural areas of the DRC, with occasional cases in other Central and West African countries, including Cameroon, Nigeria, and Liberia. These early outbreaks were characterized by a low incidence of human-to-human transmission, with most cases resulting from direct contact with infected animals or contaminated materials like bedding or clothing [27].

With improved surveillance and reporting mechanisms in the 1990s and 2000s, there was a noticeable rise in reported cases, particularly in West Africa. This increase was likely due to several factors, including better disease recognition, ecological changes like deforestation, and increased human interactions with wildlife, facilitating the virus’s spillover from animals to humans. The West African clade of mpox, which causes less severe disease than the Central African clade, became more prominent during this period. Despite these regional outbreaks, mpox remained primarily confined to Africa until 2003, when an outbreak in the United States highlighted its potential to spread beyond endemic regions [28].

The 2003 U.S. outbreak was significant, representing the first documented cases outside Africa. Traced to the importation of infected rodents from Ghana, which transmitted the virus to prairie dogs, the outbreak resulted in 47 confirmed and probable cases across six states. This event underscored mpox’s potential for international spread through the global animal trade and emphasized the importance of monitoring zoonotic diseases that could cross borders and cause outbreaks in non-endemic regions [29].

### 3.2. Global Outbreaks and Trends (2022–2024)

The mpox outbreaks between 2022 and 2023 revealed notable regional variations in case distribution. Europe was the initial epicenter, with Spain, the United Kingdom, and Germany recording the highest number of cases [30]. In North America, the United States and Canada were the most affected, with the U.S. accounting for nearly 20% of global cases by mid-2023. Asia and Oceania also observed an increase in cases, though to a lesser extent compared to Europe and North America [31]. This distribution highlighted the vulnerability of densely populated and highly interconnected urban centers. In these regions, the majority of infections occurred among men who have sex with men (MSM), with skin-to-skin contact during sexual activities being the dominant mode of transmission [32].

In North America, the United States emerged as the primary hotspot for mpox, with a significant share of global cases by mid-2023. The spread was accelerated by high urban densities and large social gatherings, primarily affecting MSM communities. U.S. authorities implemented targeted vaccination campaigns and ramped up epidemiological surveillance. Canada, on the other hand, recorded fewer cases due to rigorous monitoring and close coordination among public health services. Both countries leveraged their robust healthcare infrastructures to limit the severity of infections, maintaining a fatality rate below 1% thanks to widespread access to treatments and vaccines [33].

Europe was the first continent hit by the outbreak, with a surge in cases in Spain, the United Kingdom, and Germany. The transmission was facilitated by high international mobility and the population density of major cities. Authorities in these countries swiftly implemented control measures, such as mass testing, contact tracing, and targeted vaccination campaigns. Spain took the lead in proactive vaccination, while the United Kingdom focused on close monitoring of infection clusters. In Germany, the response centered on raising awareness within high-risk communities. The entire region benefited from well-equipped healthcare systems, which helped limit severe complications and maintain a low fatality rate [32].

In Asia, India responded quickly following the emergence of its first cases in late 2022. The detection of initial cases prompted the government to tighten border controls, particularly at airports and ports. Containment measures were immediately deployed to prevent broader community spread. Simultaneously, research programs were launched to better understand the local specifics of virus transmission and anticipate potential evolutions. Other Asian countries, such as Japan and South Korea, adopted similar approaches but recorded far fewer cases due to pre-existing surveillance systems and rapid anticipation [32,33].

In Africa, national responses varied significantly by country. In Djibouti, authorities swiftly reacted to control isolated detected cases. Enhanced epidemiological surveillance measures were implemented, accompanied by awareness campaigns to prevent any major outbreak [31]. Morocco, on the other hand, opted for a more proactive strategy by quarantining suspected cases and establishing strict epidemiological monitoring. Close collaboration with international organizations enabled the efficient coordination of prevention and control efforts. However, in other parts of Africa, insufficient healthcare infrastructure and delayed responses exacerbated complications, including secondary bacterial infections and severe symptoms.

These diverse responses underscore that adaptability and international coordination are essential for containing the spread of mpox. The development of targeted local strategies, strengthening of surveillance systems and response capacities, as well as equitable access to vaccines and treatments are essential components to mitigate the impact of future outbreaks. Increased collaboration between regions, supported by exchanges of expertise and resources, remains indispensable for bolstering global resilience against this threat.

The virus’s persistence in some areas in 2024 suggested that while significant progress was made in controlling the outbreaks, challenges remained, particularly in ensuring equitable access to vaccines and treatments. Ongoing surveillance, research into viral evolution, and public health preparedness were deemed important to prevent future outbreaks and mitigate mpox’s impact on global health [34] (Supplementary Figures S3 and S4; Supplementary Tables S1 and S2).

### 3.3. Transmission Patterns

The transmission patterns of mpox have evolved significantly, particularly with the resurgence of the virus in recent years. Mpox was primarily recognized as a zoonotic disease transmitted from animal reservoirs to humans [35]. The primary animal reservoirs for mpox are believed to be small mammals, particularly rodents, which serve as a natural host for the virus [35]. In Central and West Africa, where the disease is endemic, humans are often exposed to the virus through direct contact with infected animals, either through hunting, handling, or consuming bushmeat [35]. Studies have identified several species, including African squirrels, Gambian pouched rats, and dormice, as potential reservoirs. These animals harbor the virus and can transmit it to humans through bites, scratches, or direct contact with bodily fluids or lesions [35]. Zoonotic transmission of mpox usually occurs in rural areas with more frequent human–wildlife interactions [36]. The virus can also be transmitted through contact with contaminated materials, such as bedding or clothing, that have been in contact with an infected animal [36]. This transmission has been the primary driver of mpox cases in endemic regions, with sporadic outbreaks occurring when humans come into close contact with wildlife or when there is a spillover event from animals to humans [36]. Despite these risks, human-to-human transmission was historically considered less efficient, with transmission occurring mainly through direct contact with infected animals rather than between humans [36].

However, the recent global outbreaks have highlighted a shift in the transmission dynamics of mpox, with a notable increase in human-to-human transmission [37]. This transmission mode has become increasingly significant, particularly in urban areas and among populations with close physical contact [37]. The primary mechanism of human-to-human transmission is through direct contact with the lesions, bodily fluids, or respiratory droplets of an infected person [37]. Close physical contact, including sexual activity, has emerged as a significant risk factor during the 2022–2024 outbreaks, particularly within specific social networks [37]. Transmission through respiratory droplets is also possible, mainly when prolonged face-to-face contact occurs, although this is less common than direct contact with lesions [37].

The recent outbreaks have shown that mpox can spread efficiently within households and healthcare settings, where close and prolonged contact is common [38]. The virus can also be transmitted through fomites, with contaminated surfaces or objects serving as a vehicle for the virus to spread to individuals who touch these surfaces and subsequently touch their face or an open wound [38]. This increased efficiency in human-to-human transmission has raised concerns about the potential for mpox to establish more widespread outbreaks, particularly in densely populated areas where close contact is inevitable [38]. The evolving transmission patterns underscore the importance of early detection, isolation of cases, and the implementation of strict infection control measures to prevent the further spread of the virus [38] (Supplementary Figures S5 and S6; Supplementary Tables S3 and S4).

### 3.4. High-Risk Populations and Susceptibility

The recent global mpox outbreaks have emphasized the need to identify and understand which populations are at heightened risk to implement effective public health interventions. During the 2022–2024 outbreaks, men who have sex with men (MSM), healthcare workers, and immunocompromised individuals were disproportionately affected. These groups exhibited increased susceptibility to infection and complications, necessitating targeted strategies to minimize transmission and mitigate the disease’s impact.

MSM emerged as a particularly vulnerable population during these outbreaks, primarily due to the transmission patterns within close social and sexual networks. Epidemiological data revealed a significant proportion of cases among MSM, often associated with events or venues facilitating close physical contact. This clustering underscored the role of intimate contact, including sexual activity, in viral dissemination, a departure from the predominantly zoonotic transmission seen in earlier outbreaks. Consequently, public health authorities prioritized vaccination and education initiatives within the MSM community to curb the virus’s spread [39].

Healthcare workers also faced elevated risks due to frequent exposure to infected individuals and contaminated materials. During the outbreaks, those caring for mpox patients experienced an increased risk of infection, especially in settings with inadequate personal protective equipment (PPE) or suboptimal infection control practices. Transmission typically occurs through contact with lesions, bodily fluids, or contaminated surfaces. Despite the availability of vaccines offering cross-protection, healthcare workers remained significantly burdened, particularly in regions with large outbreaks. Stringent infection control protocols, encompassing appropriate PPE use, regular training, and vaccination, were emphasized to safeguard healthcare workers and prevent nosocomial transmission [40].

Immunocompromised individuals, including those with HIV/AIDS or cancer or organ transplant recipients, constitute another group facing heightened risks of severe mpox outcomes. These individuals are more prone to prolonged viral shedding, severe disease manifestations, and complications like secondary bacterial infections and disseminated skin lesions. They often present with atypical or more severe clinical features, and complicating diagnosis and management. The outbreaks underscored the necessity for the early identification and proactive management of mpox in immunocompromised populations to avert severe morbidity and mortality. This entails timely antiviral therapy, close monitoring, and supportive care to mitigate risks [41].

Identifying these high-risk populations has been pivotal in shaping public health responses. Targeted vaccination campaigns, enhanced infection control, and focused health messaging have been instrumental in reducing transmission and protecting vulnerable groups. Continued research into the factors influencing susceptibility in these populations is imperative in refining prevention and treatment strategies, ensuring that their needs are met in future outbreaks [42].

## 4. Clinical Features and Disease Progression

### 4.1. Incubation Period and Early Symptoms

The incubation period of mpox typically ranges from 5 to 21 days, with most cases presenting symptoms within 7 to 14 days after exposure [43]. This period reflects the time it takes for the virus to replicate within the host before clinical manifestations become evident. The length of the incubation period can vary depending on several factors, including the route of transmission, the viral load, and the host’s immune status. During this time, the virus is actively multiplying, but the infected individual remains asymptomatic and is not usually contagious, although viral shedding may begin towards the end of this phase.

Early symptoms of mpox are generally non-specific, resembling those of other febrile illnesses. This can complicate early diagnosis, especially in non-endemic regions where healthcare providers may be less familiar with the disease [44]. The initial clinical presentation typically includes fever, intense headache, muscle aches (myalgia), and back pain.

These symptoms are often accompanied by profound fatigue and malaise, which can be debilitating. Lymphadenopathy, characterized by swollen and tender lymph nodes, is a distinguishing feature of mpox that helps differentiate it from other similar illnesses, such as smallpox or chickenpox [45]. Lymphadenopathy generally occurs in the cervical, submandibular, inguinal, and axillary regions and can be quite pronounced, often preceding the appearance of the characteristic rash by a few days.

As the early symptoms progress, patients typically develop a rash, which is considered the hallmark of mpox [45]. This rash usually begins within 1 to 3 days following the onset of fever and initially appears as macules—small, flat, discolored spots on the skin. The rash quickly evolves into papules (raised lesions), vesicles (fluid-filled blisters), and then pustules (pus-filled lesions) before crusting over and eventually falling off. The rash often spreads in a centrifugal pattern, starting on the face before spreading to other body parts, including the palms of the hands and soles of the feet. In some cases, lesions may appear on mucous membranes, including the mouth and genital areas, which can be particularly painful and prone to secondary infections. The total duration of the rash phase can last for 2 to 4 weeks, during which, the patient remains contagious until all the scabs have fallen off and new skin has formed.

Understanding mpox’s incubation period and early symptoms is critical for the early detection and isolation of cases, particularly in outbreak settings where the rapid identification of infected individuals can prevent further transmission [46]. The non-specific nature of the early symptoms necessitates a high index of suspicion among healthcare providers, especially when treating patients with a history of travel to endemic areas or contact with known cases. The early recognition and differentiation from other febrile illnesses are essential for implementing appropriate infection control measures and initiating supportive care to mitigate the severity of the disease.

### 4.2. Clinical Manifestations and Complications

The clinical manifestations of mpox are primarily characterized by the development of distinctive skin lesions and a range of systemic symptoms that can vary in severity depending on the patient’s immune status and the presence of any underlying conditions [47]. The progression of the skin lesions follows a well-defined sequence, beginning as macules that evolve into papules, vesicles, and pustules before finally crusting over. These lesions are typically deep-seated, firm, and often umbilicated, resembling those seen in smallpox. The lesions appear simultaneously across the body, following a centrifugal distribution pattern, which is more concentrated on the face, palms, and soles but can also involve the trunk and extremities. In severe cases, the lesions can become confluent, leading to extensive areas of skin involvement that are prone to secondary bacterial infections, particularly in immunocompromised patients.

Systemic symptoms often accompany the cutaneous manifestations, including fever, severe headache, back pain, myalgia, and intense asthenia (weakness). Lymphadenopathy, a key clinical feature distinguishing mpox from similar diseases like smallpox, typically occurs in the cervical, submandibular, axillary, and inguinal regions [48]. This swelling of lymph nodes often precedes the rash and can persist throughout the disease. The severity of systemic symptoms can vary, with some patients experiencing only mild discomfort while others may suffer from significant debilitation that requires hospitalization.

Mpox can also lead to a range of complications, some of which can be life-threatening, particularly in vulnerable populations such as children, pregnant women, and immunocompromised individuals [49]:

- Neurological complications, although rare, can include encephalitis, seizures, and focal neurological deficits, which may result from the virus’s ability to invade the central nervous system. These complications often require intensive supportive care and can lead to long-term neurological sequelae.
- Ocular complications are another serious concern, with patients potentially developing conjunctivitis, keratitis, and, in severe cases, corneal scarring, which can lead to permanent vision loss if not promptly treated.
- Respiratory complications, such as bronchopneumonia, can occur when the virus spreads to the respiratory tract, either through direct viral invasion or secondary bacterial infection, and are a leading cause of mortality in severe cases of mpox.

The potential for these complications highlights the need for early recognition and management of mpox, particularly in high-risk patients [50]. Supportive care is the mainstay of treatment, focusing on relieving symptoms and preventing secondary infections. In severe cases, antiviral therapy with agents such as tecovirimat may be considered, although its use is currently limited and primarily recommended for patients with severe or complicated diseases. The prevention of complications requires a multidisciplinary approach, including the following:

- The management of skin lesions to prevent bacterial superinfection;
- Monitoring for signs of neurological or respiratory involvement;
- Providing appropriate ophthalmologic care when ocular manifestations are present (Supplementary Figures S7 and S8; Supplementary Table S5).

The early stages of mpox clinical disease, particularly its initial cutaneous manifestations, can often be mistaken for other infections such as varicella or herpes simplex virus (HSV). Differentiating between these conditions is essential due to the clinical similarities, which include vesicular and pustular lesions. On the skin, early mpox lesions can mimic those of varicella, which also presents with vesicles and papules. Moreover, early genital lesions of mpox may closely resemble those caused by HSV-1 or HSV-2, making a differential diagnosis critical to prevent misidentification. Therefore, a thorough clinical history, coupled with laboratory tests like PCR, is pivotal to accurately distinguishing between these diseases in the affected regions [49,50].

### 4.3. Outcomes and Mortality Rates

The outcomes of mpox infection can vary widely, ranging from complete recovery to severe complications and, in some cases, death [51]. Most individuals infected with mpox recover fully after the acute phase of the illness, which typically lasts between two to four weeks. However, the disease can result in long-term sequelae, particularly in patients who experience severe or complicated infections [51]. These sequelae can include scarring from skin lesions, ocular damage leading to vision loss, and, in rare cases, neurological impairments that may persist long after the infection has resolved [51]. The extent of long-term effects is influenced by the initial infection’s severity and complications during the disease course [51].

The mortality rates associated with mpox have historically varied depending on the clade of the virus involved and the healthcare context in which patients are treated [52]. The Central African (Congo Basin) clade of mpox is associated with a higher case fatality rate (CFR), historically estimated to be between 10% and 15%, particularly in regions with limited access to healthcare [53]. In contrast, the West African clade, which was more prevalent in recent global outbreaks, generally exhibits a lower CFR, typically ranging from 1% to 3% [53]. However, even with the less virulent West African clade, mortality can still occur, particularly in vulnerable populations such as young children, pregnant women, and immunocompromised individuals, who are more susceptible to severe disease and complications [53].

Several factors influence mpox severity, outcomes, and mortality rates [54]. Age is a key determinant, with young children and older adults more prone to severe disease [54]. The presence of underlying health conditions, such as HIV/AIDS or other forms of immunosuppression, also plays a critical role in determining disease severity [54]. Immunocompromised individuals are at higher risk for severe manifestations, prolonged illness, and death, primarily due to their reduced ability to mount an effective immune response against the virus [54]. Additionally, the extent and quality of medical care available to the patient are essential in managing the disease and reducing mortality [54]. Access to supportive care, including hydration, nutrition, and treatment of secondary infections, can significantly improve outcomes, while the lack of such care can lead to higher mortality rates [54].

The availability of antiviral treatments, such as tecovirimat, has shown promise in reducing the severity of symptoms and improving outcomes, particularly in severe cases or those involving high-risk patients [55]. However, the use of these treatments is still limited and often reserved for the most severe cases, highlighting the need for further research and broader access to effective antiviral therapies [55]. Public health measures, such as vaccination strategies with the smallpox vaccine that provides cross-protection against mpox, have been essential in mitigating the disease’s impact, especially during outbreaks [55]. Understanding the factors contributing to the variability in outcomes and mortality rates is essential for developing targeted interventions to reduce the burden of mpox. Ongoing surveillance, improved access to healthcare, and the continued development of antiviral therapies and vaccines are critical components in mitigating the effects of future outbreaks and improving patient outcomes across diverse populations.

## 5. Diagnosis of Mpox

### 5.1. Specimen Collection and Laboratory Techniques

The accurate diagnosis of mpox hinges on properly collecting clinical specimens and applying appropriate laboratory techniques. Specimen collection is a critical first step, and it requires careful adherence to guidelines to ensure the accuracy and reliability of the test results [56]. The primary specimens collected for diagnosis are from skin lesions, which are the most characteristic and accessible source of viral material [56]. These lesions progress through several stages—macules, papules, vesicles, pustules, and scabs—each of which can be sampled depending on the stage of the disease at the time of presentation [56]. The ideal specimens include swabs of the vesicular or pustular fluid and crusts from healing lesions, which contain high concentrations of the virus [56].

When collecting specimens from skin lesions, it is essential to use a sterile technique to avoid contamination [56]. A sterile, dry swab is typically used to collect fluid from a vesicle or pustule [56]. For crusted lesions, the scab can be gently lifted using a sterile instrument, and the underlying tissue can be swabbed [56]. The swabbed material is then placed in a sterile, leak-proof container, such as a viral transport medium (VTM), to preserve the virus during transport to the laboratory [56]. In addition to lesion swabs, other biological materials such as blood, throat swabs, and, in some cases, urine may be collected to support diagnosis, particularly in cases with systemic symptoms or in patients who present with atypical features [52]. These samples offer additional insights into viral spread and are useful when lesion sampling is not feasible [52]. The laboratory diagnosis of mpox predominantly relies on molecular methods, with polymerase chain reaction (PCR) being considered the gold standard technique due to its high sensitivity and specificity [52]. PCR is used to detect the viral DNA of the mpox virus, making it an essential tool for confirming the presence of the infection [57]. The targets of the PCR assay are specific genes within the virus, typically focusing on the conserved regions of the *Orthopoxvirus* genome, such as the DNA polymerase gene (E9L) or the hemagglutinin gene (A27L) [57]. This approach not only identifies mpox but also distinguishes it from other *Orthopoxviruses*, such as vaccinia or cowpox, which may present similar clinical features but require different therapeutic approaches [57]. Thus, this technique provides both diagnostic precision and differentiation, bridging molecular and clinical diagnostic needs.

In addition to PCR, electron microscopy and viral culture can be supplementary diagnostic tools. However, they are less commonly employed due to the time, expertise, and biosafety requirements [58]. Electron microscopy can visually confirm the virus by revealing its characteristic brick-shaped virions. Still, it lacks the sensitivity of PCR and is typically reserved for cases where rapid molecular diagnostics are unavailable [58]. Viral culture, while definitive, is time-consuming and requires high-level biosafety facilities (BSL-3), making it less practical for routine diagnosis [58]. Serological tests, such as enzyme-linked immunosorbent assays (ELISA) to detect anti-*Orthopoxvirus* antibodies, can be helpful in retrospective diagnosis or epidemiological studies. Still, they are not typically used for acute diagnosis due to cross-reactivity with other *Orthopoxviruses* and the delayed appearance of antibodies following infection [58].

The success of diagnosing mpox depends on the appropriate selection of specimens and laboratory techniques and the timely collection and processing of samples. Delays in sample collection or improper handling can result in the degradation of viral DNA, leading to false-negative results. Therefore, healthcare providers must be trained in the correct specimen collection, storage, and transport procedures to ensure that the laboratory results accurately reflect the patient’s infection status. A proper diagnosis is important for guiding clinical management, implementing infection control measures, and preventing the spread of the virus within the community (Supplementary Table S6).

### 5.2. Molecular Methods

Molecular diagnostics, especially PCR-based methods, form the cornerstone for diagnosing mpox, with PCR being the gold standard due to its ability to detect the virus with high accuracy [57]. PCR assays enable precise identification even when the viral load is low, allowing for early detection and confirmation of mpox infection [59]. Among these, real-time PCR (qPCR) is the most widely used technique in clinical settings due to its capacity to quantify viral DNA in real-time, providing insights into the viral load and disease progression [59]. By targeting conserved genetic regions of the *Orthopoxvirus* genome, such as the E9L DNA polymerase gene or the A27L hemagglutinin gene, qPCR ensures accurate differentiation between mpox and other *Orthopoxviruses* (e.g., vaccinia or cowpox), which can present similar symptoms but require distinct clinical management strategies [59]. This overlap in diagnostic and molecular methodologies highlights the integrated role of PCR in both identifying and managing mpox infections.

Multiplex PCR is another valuable technique in diagnosing mpox, mainly when rapid differentiation from other pathogens is necessary [60]. This method allows for the simultaneous amplification of multiple targets in a single reaction, making it possible to identify co-infections or distinguish between different viral strains [60]. For example, a multiplex PCR assay can be designed to detect several *Orthopoxvirus* species in addition to the mpox virus, providing comprehensive diagnostic information from a single test [60]. The ability to conduct multiplex assays is instrumental in outbreak scenarios, where speed and accuracy are critical for guiding public health interventions [60].

Emerging technologies like Loop-Mediated Isothermal Amplification (LAMP) and Recombinase Polymerase Amplification (RPA) are also gaining attention as potential tools for mpox diagnosis [61]. These methods offer the advantage of rapid and straightforward detection without sophisticated laboratory equipment, making them suitable for use in resource-limited settings [61]. LAMP amplifies DNA at a constant temperature, eliminating the need for thermal cycling, and can produce results within 30 min [61]. This method can be used at the point of care, which is essential during outbreaks in remote or underserved areas [61]. RPA, similarly, is an isothermal technique that operates at a lower temperature and can be completed in 20 to 30 min [62]. LAMP and RPA have shown promise in detecting the mpox virus, particularly for field diagnostics where traditional PCR equipment is unavailable [61].

Integrating these molecular methods into diagnostic workflows is critical for improving the speed and accuracy of mpox detection [62]. While traditional PCR remains the benchmark for laboratory confirmation, developing portable, rapid diagnostic tests using technologies like LAMP and RPA could revolutionize outbreak responses, particularly in regions with limited access to laboratory infrastructure [62]. These emerging technologies expand diagnostic capabilities and enhance the ability to conduct large-scale screening and surveillance, which are essential for controlling the spread of mpox and other emerging infectious diseases [62]. As these methods evolve, their application in clinical and field settings will likely increase, providing more flexible and responsive diagnostic options for healthcare providers worldwide [62] (Supplementary Table S7).

## 6. Treatment and Management Strategies

### 6.1. Supportive Care

Supportive care is the cornerstone of treatment for mpox, with a focus on alleviating symptoms, managing pain, and ensuring adequate nutritional support to aid in the recovery process [63]. The clinical management of mpox involves addressing the various symptoms that arise during the illness, ranging from fever and rash to more severe systemic symptoms [63]. Pain management is critical, as the skin lesions associated with mpox can be extremely painful, mainly when they occur on sensitive areas such as the face, genitals, or mucous membranes [63]. Nonsteroidal anti-inflammatory drugs (NSAIDs) and acetaminophen are commonly used to reduce fever and manage mild to moderate pain [63]. In cases of severe pain, mainly when lesions are widespread or involve deeper tissue layers, more potent analgesics, including opioids, may be necessary to provide adequate relief and improve the patient’s comfort [63].

In addition to pain management, ensuring proper hydration and nutritional support is critical, particularly for patients with extensive skin involvement that can lead to significant fluid loss [64]. Oral rehydration solutions or intravenous fluids may be required to maintain electrolyte balance and prevent dehydration, especially in children and those with severe disease [64]. Nutritional support plays a vital role in the recovery process, as malnutrition can exacerbate the severity of the illness and delay healing [64]. Patients are encouraged to maintain a balanced diet rich in proteins and essential vitamins for skin repair and immune function [64]. In cases where oral intake is insufficient due to painful oral lesions or systemic symptoms like nausea and vomiting, enteral feeding or parenteral nutrition may be necessary to meet the patient’s dietary needs [64].

Monitoring for and managing potential complications is another critical aspect of supportive care [65]. Secondary bacterial infections are a common complication of mpox, particularly in cases where the skin barrier is compromised by extensive or ulcerated lesions [65]. Prophylactic antibiotics are not routinely recommended but should be considered in patients showing signs of secondary infection, such as increased erythema, pus formation, or systemic signs of infection like fever and elevated white blood cell counts [65]. Antiseptic solutions for wound care can help reduce the risk of infection and promote faster healing of lesions [65]. Additionally, for patients with respiratory symptoms, supportive care may include the use of supplemental oxygen or, in severe cases, mechanical ventilation to manage respiratory distress [65].

Managing the psychological impact of mpox is also an essential component of supportive care [66]. The disfiguring nature of the skin lesions, combined with the social stigma associated with the disease, can lead to significant emotional and psychological stress [66]. Counselling and psychological support should be offered to help patients cope with the anxiety, depression, or social isolation that may arise during and after their illness [66]. Providing education about the disease, including its natural course and expected outcomes, can help alleviate some of the fears and misconceptions that contribute to psychological distress [66].

Effective supportive care requires a multidisciplinary approach that addresses patients’ physical, nutritional, and psychological needs [66]. This approach is vital for reducing morbidity and improving overall outcomes and quality of life for individuals affected by mpox [66]. As research into mpox continues, these supportive care strategies may evolve, but they will remain a fundamental aspect of the clinical management of the disease [66] (Supplementary Table S8).

### 6.2. Antiviral Therapies

The treatment of mpox has increasingly focused on the use of antiviral therapies, particularly in cases involving severe disease or high-risk populations. Tecovirimat, brincidofovir, and cidofovir are the primary antiviral agents that have been explored for their efficacy against the mpox virus [67]. Each has distinct mechanisms of action, efficacy profiles, and potential side effects.

Tecovirimat, or TPOXX, is mpox’s most widely recommended antiviral [67]. It targets the viral protein p37, which is important for forming and disseminating extracellular virions, effectively inhibiting viral spread within the host [67]. Clinical trials and compassionate use cases have demonstrated that tecovirimat significantly reduces the duration of viral shedding and accelerates the resolution of symptoms, particularly skin lesions [67]. Its safety profile is generally favorable, with the most commonly reported side effects being mild, such as headache and nausea [67]. However, due to its relatively recent approval, long-term safety data are still being collected, and its use is primarily recommended for patients with severe disease or those at high risk of complications, such as immunocompromised individuals [67].

Brincidofovir, a lipid-conjugated prodrug of cidofovir, is another promising antiviral in treating mpox [68]. It inhibits viral DNA polymerase, thereby preventing viral replication [68]. Brincidofovir offers a more favorable safety profile than cidofovir, particularly regarding nephrotoxicity, which has been a significant concern with cidofovir [68]. However, its use in mpox remains limited, primarily due to the need for extensive clinical trial data specifically for this indication [68]. Early studies suggest that brincidofovir may reduce the severity of mpox symptoms and prevent disease progression. Still, more research is needed to confirm its efficacy and safety in larger cohorts [68]. The side effects of brincidofovir can include gastrointestinal disturbances, such as nausea, vomiting, and diarrhea, as well as potential liver enzyme elevations, which necessitate regular monitoring during treatment [68].

Cidofovir, the parent compound of brincidofovir, has been used for many years to treat various DNA virus infections, including those caused by poxviruses [69]. It is a potent inhibitor of viral DNA polymerase, but its use is limited by its significant nephrotoxicity [69]. Cidofovir is administered intravenously, typically in conjunction with probenecid and saline hydration, to mitigate its renal toxicity [69]. Despite its effectiveness against *Orthopoxviruses*, including mpox, the risk of kidney damage has restricted its use to cases where other treatments are unavailable or contraindicated [69]. The side effects of cidofovir are well documented and include not only nephrotoxicity but also neutropenia, uveitis, and potential teratogenic effects, which require careful consideration and patient monitoring [69].

The use of these antiviral therapies in treating mpox is guided by the severity of the disease, the patient’s risk factors, and the availability of the drugs. While tecovirimat is often preferred due to its targeted mechanism and favorable safety profile, brincidofovir and cidofovir remain important options, particularly in complex cases where other treatments may not be suitable [69]. Ongoing research and clinical trials are critical to elucidate the optimal use of these antivirals, including their efficacy in different patient populations and the potential for combination therapy to enhance treatment outcomes (Supplementary Figure S9).

### 6.3. Immunotherapy and Vaccination

Immunotherapy and vaccination are essential in managing and preventing mpox, especially in severe cases and high-risk groups. Vaccinia Immune Globulin Intravenous (VIGIV) is a critical immunotherapeutic option for severe mpox, particularly in immunocompromised patients or those at high risk of complications [70]. VIGIV, a preparation of immune globulin with high titers of antibodies against the vaccinia virus, is an adjunctive treatment when antivirals alone may not suffice. It neutralizes the virus, reducing the viral load and controlling the infection’s spread [70]. Its use is generally limited to severe or life-threatening cases and individuals with contraindications to the smallpox vaccine or complications from live virus vaccines like ACAM2000. VIGIV has been shown to decrease mortality and improve outcomes in severe mpox, although its availability is limited, and it is typically used with antiviral therapies [70].

Vaccination remains essential for mpox prevention, especially for outbreak control and protecting high-risk groups like healthcare workers and close contacts of infected individuals. Two primary vaccines are used: MVA-BN (Modified Vaccinia Ankara–Bavarian Nordic), Jynneos or Imvanex, and ACAM2000 [71,72]. MVA-BN, a third-generation vaccine, uses a highly attenuated, non-replicating form of the vaccinia virus. It is approved for preventing smallpox and mpox and is favored for its improved safety, especially in immunocompromised individuals [71]. MVA-BN is administered in two doses and induces a robust immune response, providing cross-protection against mpox with fewer adverse effects than earlier vaccines. It has been useful in recent outbreaks, with mass vaccination campaigns helping contain the virus’s spread [71].

ACAM2000 is a second-generation vaccine developed from a live, replicating strain of the vaccinia virus [72], specifically designed to provide strong immunity against smallpox. However, due to its replicating nature, it carries an increased risk of serious adverse effects such as myocarditis, pericarditis, and complications in immunocompromised individuals or those with skin conditions like eczema [72]. Administered as a single dose using a bifurcated needle, this vaccine is primarily utilized in contexts where rapid immunization is essential, such as post-exposure prophylaxis or in military settings [72].

Vaccine availability varies regionally, with MVA-BN more accessible in Europe and North America, while ACAM2000 is often stockpiled for emergencies. Research into next-generation vaccines aims to improve safety and efficacy, making them more accessible to broader populations. In mpox, vaccination is critical for individual protection and achieving herd immunity in at-risk communities, thus reducing overall transmission [71] (Supplementary Table S9).

## 7. Prevention and Public Health Implications

### 7.1. Vaccination Strategies and Efficacy

Vaccination remains a cornerstone of public health strategies to prevent mpox outbreaks, particularly in regions where the virus poses a significant threat. The effectiveness of vaccination campaigns has been demonstrated in various settings, both during historical smallpox eradication efforts and more recent mpox outbreaks [73]. The primary preventive vaccines include MVA-BN (Modified Vaccinia Ankara–Bavarian Nordic), also known as Jynneos or Imvanex, and ACAM2000, a second-generation smallpox vaccine [73,74]. These vaccines have been critical in controlling the spread of mpox, particularly during the global outbreaks observed in the early 2020s [73].

MVA-BN is a third-generation vaccine that has gained prominence due to its favorable safety profile, especially among populations with contraindications to traditional live-virus vaccines, such as immunocompromised individuals [73]. The non-replicating nature of MVA-BN reduces the risk of serious adverse effects, making it a preferred choice in large-scale vaccination campaigns. Studies have shown that MVA-BN is highly effective in eliciting a robust immune response that provides cross-protection against mpox, significantly reducing the incidence of disease among vaccinated individuals [73]. In outbreak scenarios, MVA-BN has been used prophylactically and as post-exposure prophylaxis (PEP) to curb the spread of the virus among close contacts and healthcare workers.

ACAM2000, derived from the same vaccinia strain, is also known for its effectiveness against other *Orthopoxviruses*, such as mpox [74]. Although its use is limited in certain populations due to potential risks, it remains a strategic asset in emergency stockpiles. It is prioritized in situations where a rapid immune response is needed to contain outbreaks and protect frontline responders, making it a valuable tool for managing critical scenarios. The vaccine’s efficacy in preventing mpox has been well documented, with studies indicating that it can provide long-lasting protection, reducing both the severity and spread of the disease during outbreaks [74]. However, carefully screening recipients is necessary to minimize the risk of vaccine-related complications.

The effectiveness of vaccination campaigns depends not only on the efficacy of the vaccines themselves but also on the strategies employed to deploy them. During the 2022–2024 mpox outbreaks, vaccination campaigns were strategically targeted at high-risk populations, including men who have sex with men (MSM), healthcare workers, and individuals with known exposure to confirmed cases [75]. These targeted approaches were essential in reducing the overall transmission of the virus and preventing more significant outbreaks. Public health authorities utilized ring vaccination strategies, where immediate contacts of confirmed cases were vaccinated to create a buffer of immune individuals, effectively containing the virus within specific communities.

Furthermore, the role of vaccination in achieving herd immunity is critical in the long-term control of mpox [76]. High vaccination coverage within at-risk populations can significantly reduce the virus’s ability to spread, thus lowering the overall disease incidence. However, ensuring equitable access to vaccines remains challenging, particularly in resource-limited regions where the healthcare infrastructure is often inadequate to support large-scale vaccination efforts. Global cooperation and the equitable distribution of vaccines are essential to preventing the resurgence of mpox in both endemic and non-endemic regions.

The ongoing evaluation of vaccination strategies and their effectiveness is vital for refining the public health responses to mpox. Continued research into vaccine efficacy, particularly in diverse population groups, will help optimize vaccination protocols and improve outcomes during future outbreaks. As new vaccines are developed and existing ones are adapted, vaccination will continue to play a central role in the global effort to control mpox and prevent its spread (Supplementary Figure S8).

### 7.2. Public Health Policies and Recommendations

The global response to the mpox outbreak has been shaped by comprehensive public health policies and recommendations from organizations such as the World Health Organization (WHO) and national health authorities. These guidelines have been instrumental in coordinating the international efforts to prevent and control mpox outbreaks, especially in light of the recent global resurgence of the virus. The WHO’s guidelines provide a framework for monitoring, diagnosing, and managing mpox. They emphasize the importance of early detection and a rapid response to outbreaks by implementing robust surveillance systems, particularly in regions where the virus is endemic or at risk of importation. This framework includes laboratory-based diagnostics such as PCR testing to confirm cases and contact tracing to identify and monitor individuals potentially exposed to the virus. Moreover, the WHO stresses the importance of public education campaigns to raise awareness about mpox transmission modes and the preventive measures that can reduce the risk of infection [77].

National public health responses have varied significantly depending on the local context and available resources. In high-income countries, strategies have primarily focused on vaccination campaigns targeting high-risk populations such as healthcare workers, men who have sex with men (MSM), and individuals with known exposure to confirmed cases. These efforts have been supported by vaccine stockpiles such as those for MVA-BN and ACAM2000, which have been deployed to prevent the spread of the virus. Alongside vaccination, national health authorities have also issued guidance on using antiviral therapies like tecovirimat for treating severe cases, as well as implementing isolation and infection control measures to prevent nosocomial transmission. In contrast, low- and middle-income countries, particularly in Africa where mpox is endemic, have faced significant challenges in implementing the WHO recommendations due to limited resources and healthcare infrastructure. These countries have often relied on more traditional public health measures, such as quarantine and movement restrictions, to control outbreaks. However, the lack of access to vaccines and antivirals has made it difficult to contain the virus, leading to higher morbidity and mortality rates. The WHO has called for increased international support to help these countries strengthen their surveillance and response capacities, including providing vaccines, antivirals, and diagnostic tools [4]. Global cooperation is essential to ensure that all countries can respond effectively to mpox outbreaks and prevent the virus from spreading to new regions [78].

While high-income countries have been able to implement comprehensive vaccination and treatment strategies, low- and middle-income countries require significantly more support to achieve similar outcomes. The WHO’s role in providing guidance and facilitating international cooperation is important to ensuring that all countries can effectively respond to mpox and prevent future outbreaks. It is also essential to note that neither previous mpox infections nor past vaccinations always prevent future infections, highlighting the need to maintain preventive and control measures, even in vaccinated or previously exposed populations [79].

### 7.3. Future Directions in Prevention and Control

As the global health community reflects on the lessons learned from the 2022–2023 mpox outbreak, there is a growing consensus on the need to enhance surveillance, outbreak responses, and vaccination strategies to prevent and control future outbreaks [80]. Effective prevention and control of mpox will require a multifaceted approach that addresses the weaknesses identified during the recent epidemic while leveraging new technologies and strategies.

One of the primary recommendations for improving mpox prevention and control is strengthening global surveillance systems [80]. The 2022–2023 outbreak highlighted significant gaps in the timely detection and reporting of cases, particularly in regions where mpox was not previously considered a significant threat [80]. To address these gaps, there is a need for enhanced surveillance that includes both endemic and non-endemic regions. This can be achieved by integrating mpox surveillance into broader infectious disease monitoring frameworks, using advanced data analytics and real-time reporting systems to identify and respond to emerging threats. Additionally, improving the laboratory capacity in low- and middle-income countries is essential for accurate and timely diagnosis, which is essential for effective outbreak management [80].

Another critical area for future improvement is the global response to outbreaks [81]. The 2022–2023 outbreak underscored the importance of rapid and coordinated international action to contain the virus [81]. To enhance outbreak response, countries should prioritize the development of national preparedness plans that include clear protocols for case management, contact tracing, and the deployment of medical countermeasures [81]. These plans should be regularly updated and tested through simulations to ensure readiness for future outbreaks [81]. Furthermore, international cooperation, facilitated by organizations like the World Health Organization (WHO), will be essential for coordinating cross-border responses and ensuring that resources are allocated efficiently to needy areas [81].

Vaccination strategies must also be refined and expanded to ensure broader protection against mpox [82]. The recent outbreak revealed the limitations of the current vaccination coverage, particularly in high-risk populations [82]. Future strategies should focus on expanding access to vaccines, particularly in low- and middle-income countries where the disease burden is often most significant [82]. This includes increasing the production and distribution of vaccines like MVA-BN and ACAM2000, as well as developing new vaccines that are safer and more effective across diverse populations [82]. Targeted vaccination campaigns, especially those aimed at high-risk groups, should be part of a comprehensive strategy that includes public education and community engagement to increase vaccine uptake and build trust in vaccination programs [82].

In addition to these immediate priorities, there is a need for ongoing research to understand the epidemiology of mpox better and to develop new tools for prevention and control. This includes studies on the long-term efficacy of vaccines, the development of antiviral therapies, and the exploration of novel immunotherapies. Research should also focus on understanding the socio-economic and environmental factors contributing to the spread of mpox, particularly in endemic regions, to inform more effective public health interventions.

The future of mpox prevention and control will depend on the global community’s ability to learn from past experiences and to implement more robust, equitable, and innovative approaches. By improving surveillance, enhancing outbreak responses, refining vaccination strategies, and investing in research, we can better protect populations around the world from the threat of mpox and other emerging infectious diseases.

## 8. Conclusions

The resurgence of mpox underscores the dynamic nature of infectious diseases and the critical need for global preparedness. While advancements in diagnostics and therapeutics offer hope, significant challenges remain. The evolving transmission patterns and potential for increased virulence due to mutations highlight the necessity of heightened vigilance and proactive public health measures. The 2022–2023 outbreak emphasized the importance of equitable access to vaccines and treatments, along with the need for robust surveillance and rapid response capabilities in both endemic and non-endemic regions. In light of these challenges, the World Health Organization (WHO) has extended an invitation to pharmaceutical companies producing the mpox vaccine to submit an expression of interest for emergency use as of 7 August 2024. This initiative aims to accelerate vaccine production, ensuring that the global supply can meet the demand during future outbreaks. Future research should prioritize the development of more effective and accessible diagnostics, the exploration of novel antiviral therapies and vaccines, and a deeper understanding of the socio-economic and environmental factors contributing to mpox transmission. By strengthening global health systems and fostering international collaboration, we can better prepare for and mitigate the impact of future mpox outbreaks, safeguarding public health on a global scale.

## Figures and Tables

**Table 1:** Timeline depicting the global detection of mpox (MPXV) in humans and animals, alongside the reported incidence of mpox cases. This illustrates the historical emergence and recent resurgence of the virus, highlighting its increasing global impact [19].

Feature	MPXV Clade I	MPXV Clade IIa	MPXV Clade IIb	VARV ^e^
Endemic Region	Central Africa ^a^	West Africa ^b^	West Africa ^c^	Eradicated
Global Outbreak	No	2003	2018–2023	Eradicated
Animal Reservoir	Multiple species	Multiple species	Multiple species	None
Vesicular Lesions (Human)	Yes	Yes	Yes	Yes
Lethality (Human)	10.6%	Low	3.6% ^d^	~35%
Select Agent	Yes	No	No	Yes
Vaccine Available (Human) ^f^	Yes	Yes	Yes	Yes
Therapeutics Available (Human) ^g^	Yes	Yes	Yes	Yes

^a^ Mainly DRC. ^b^ Ivory Coast, Liberia, Sierra Leone, Ghana, and Cameroon. ^c^ Nigeria. ^d^ Deaths in outbreak in Nigeria. ^e^ Variola virus that causes smallpox. ^f^ Smallpox vaccines Jynneos and ACAM2000. ^g^ Tecoviramat, brincidofovir, and cidofovir.

## Supplementary Materials

The following supporting information can be downloaded at: https://www.mdpi.com/article/10.3390/v16111737/s1, Figure S1: Timeline depicting the global detection of Mpox virus (MPXV) in humans and animals, alongside the reported incidence of mpox cases. This illustrates the historical emergence and recent resurgence of the virus, highlighting its increasing global impact. Figure S2: Evolutionary relationships between different Orthopoxvirus genomes, including MPXV clades I and II, Variola virus, and Vaccinia virus. The tree is based on genetic sequence data, showing branching patterns reflecting evolutionary divergence over time. Figure S3: Current geographic distribution of confirmed mpox cases, providing a visual representation of the global spread and prevalence of the disease. Figure S4: Geographic distribution of confirmed mpox cases during the January to September 2022 outbreak. Confirmed cases include laboratory-confirmed MPXV and potential orthopoxvirus cases. Data source: CDC. Diagram created using GraphPad Prism 9. Figure S5: Potential routes of mpox transmission from animals to humans illustrate various pathways through which the virus can spill over from animal reservoirs to human populations. Figure S6: Mpox from transmission to symptoms—A visual guide depicting the timeline and progression of mpox infection, from initial exposure to the development of characteristic symptoms and potential complications. Figure S7: Overview of mpox: Non-specific symptoms and potential complications, summarizing the diverse clinical manifestations of mpox, ranging from initial non-specific symptoms to potential complications affecting various organ systems. Figure S8: (A) Stages of mpox infection: A visual timeline showcasing the sequential progression of skin lesions and the duration of each stage, from macules to scabs. (B) Future directions in developing mpox vaccines, highlighting potential strategies and approaches for creating next-generation vaccines with improved safety, efficacy, and accessibility. Figure S9: Life cycle of mpox virus and antiviral mechanisms of action, illustrating the key stages of the viral life cycle and the points at which different antiviral drugs, such as Tecovirimat, Brincidofovir, and Cidofovir, exert their inhibitory effects. Table S1: Number of confirmed mpox cases and deaths reported to WHO by region between 1 January 2022, and 30 September 2023, providing a breakdown of the global mpox burden across different WHO regions. Table S2. Global mpox outbreak in 2022 by continent, presenting data on confirmed mpox cases in different continents during the 2022 outbreak. Data source: CDC, updated on 13 September 2022. Table S3. Animals naturally and experimentally infected with mpox virus, listing various animal species that have been documented to be naturally or experimentally infected with MPXV, along with relevant references. Table S4. Susceptible animal species and their classification in relation to mpox virus infection, categorizing different animal species based on their susceptibility to MPXV infection, providing a comprehensive overview of potential animal reservoirs and hosts. Table S5: Contrasting the 2022 mpox outbreak with prior outbreaks, comparing key epidemiological and clinical features between the 2022 outbreak and previous mpox outbreaks, highlighting the distinct characteristics of the recent resurgence. Table S6: Case definitions for mpox, outlining the criteria for classifying suspected, probable, and confirmed cases of mpox, aiding in the accurate identification and reporting of cases. Table S7: Comprehensive overview of diagnostic methods for mpox, summarizing various diagnostic techniques used for mpox detection, including their underlying principles, sample types, advantages, and limitations. Table S8: Supportive treatments for mpox symptoms and complications, listing supportive care measures used to manage the diverse symptoms and complications associated with mpox infection. Table S9: Candidate vaccines and antiviral drugs for the prevention and treatment of human poxvirus infections, providing a list of potential vaccines and antiviral drugs under investigation or in use for the prevention and treatment of human poxvirus infections, including mpox.
