# Supplementary material for: Mpox Resurgence: A Multifaceted Analysis for Global Preparedness
Source: Viruses. 2024 Nov 5;16(11):1737. doi: 10.3390/v16111737 (PMC11598846; doi:10.3390/v16111737)
Supplement: Supplementary file 1 [file viruses-16-01737-s001.zip › viruses-3274135-supplementary.pdf]

Supplementary Information

S.1.1 Case Studies and Special Populations

The clinical course of mpox can vary significantly among different population groups, particularly in children, pregnant women, and immunocompromised patients, who are often at higher risk for severe disease and complications [S1]. Children, due to their developing immune systems, are more vulnerable to severe manifestations of mpox [S1]. Studies have shown that children under the age of 8 are particularly susceptible to complications such as secondary bacterial infections, sepsis, and respiratory distress [S1]. The disease in children often presents with more extensive rash and systemic involvement, which can lead to prolonged recovery times and higher morbidity rates compared to adults [S1]. In addition, dehydration due to extensive skin involvement and the inability to maintain proper fluid intake can complicate the clinical course in paediatric patients, necessitating more aggressive supportive care [S1].

Pregnant women infected with mpox face unique challenges due to the potential risks to both the mother and the fetus [S2]. The virus can cross the placental barrier, leading to congenital mpox, which may result in fetal death, preterm birth, or neonatal mpox, characterized by severe systemic involvement in the newborn [S2]. Case studies have documented instances of fetal demise associated with maternal mpox, underscoring the importance of early diagnosis and management in this vulnerable group [S2]. Pregnant women with mpox may also experience more severe disease courses due to the immunological changes associated with pregnancy, which can alter the body's response to the infection [S2]. The management of mpox in pregnant women requires a multidisciplinary approach, including obstetric care, to optimize outcomes for both the mother and the child [S2].

Immunocompromised patients, including those with HIV/AIDS, cancer, or those on immunosuppressive therapies, are at significantly higher risk for severe and prolonged mpox infection [S3]. The virus can cause more extensive and persistent lesions in these patients, with a higher likelihood of secondary complications such as bacterial infections, pneumonia, and encephalitis [S3]. Immunocompromised individuals may also exhibit atypical presentations of mpox, where the classic rash may be absent or minimal, leading to challenges in diagnosis [S3]. Furthermore, viral shedding in these patients can be prolonged, raising concerns about transmission and the need for extended isolation periods [S3]. Case studies have highlighted the importance of early antiviral intervention and intensive supportive care in this population to prevent fatal outcomes [S3].

Several case studies have also documented atypical presentations of mpox, which can occur even in otherwise healthy individuals [S4]. These atypical cases may involve unusual rash locations, such as isolated lesions on the genitalia or perianal region or presentations that mimic other dermatological conditions like chickenpox or syphilis [S4]. Such cases underscore the diagnostic challenges that healthcare providers may face, particularly in non-endemic regions where mpox is less commonly seen [S4]. Identifying these atypical presentations is critical for preventing misdiagnosis and ensuring that patients receive appropriate care and isolation to prevent further virus transmission [S4].

The variability in clinical presentations and outcomes among special populations highlights the need for tailored approaches to the management of mpox. This includes heightened awareness among healthcare providers of the risks in children, pregnant women, and immunocompromised patients, as well as the potential for atypical presentations. Continued research and case study analysis are essential for improving our understanding of the disease in these vulnerable groups and for developing strategies to mitigate the impact of mpox on public health.

S.1.2 Histopathology and Electron Microscopy

Histopathological examination and electron microscopy are essential tools for diagnosing and studying mpox (MPXV) infections, providing detailed insights into the cellular and ultrastructural changes induced by the virus. The histopathological features of MPXV infection are characterized by a range of distinctive findings that reflect the virus's ability to cause extensive tissue damage and inflammation. Lesions in the skin, which are the most commonly examined tissues, typically show necrosis, accompanied by ballooning degeneration of keratinocytes [S5]. These affected cells often contain eosinophilic cytoplasmic inclusions, known as Guarnieri bodies, indicative of Orthopoxvirus replication sites [S5]. These inclusions represent viral factories where MPXV replication occurs and can be visualized using routine histological stains, making them a key diagnostic feature.

In addition to these cytopathic effects, histopathological analysis often reveals a pronounced inflammatory response characterized by the infiltration of mononuclear cells, including lymphocytes, macrophages, and plasma cells, into the affected tissues [S6]. This inflammation can lead to microabscesses, particularly in more severe or secondary infected lesions. Vasculitis, or inflammation of the blood vessels, is another hallmark of MPXV infection, which can contribute to the hemorrhagic aspects observed in some lesions [S6]. Histopathological findings may also vary depending on the stage of the lesion, with early lesions showing more pronounced viral cytopathic effects and later stages exhibiting more significant inflammatory responses and tissue repair processes.

Electron microscopy offers a powerful tool for the direct visualization of MPXV, providing high-resolution images that reveal the detailed structure of the virus [73]. MPXV, like other Orthopoxviruses, has a characteristic brick-shaped virion that measures approximately 200-250 nm in length [S7]. The virion consists of a complex structure with an outer envelope, a core containing the viral DNA, and lateral bodies that flank the core. These features can be distinctly visualized under transmission electron microscopy (TEM), often used to confirm the presence of MPXV in clinical samples, mainly when rapid molecular diagnostics are unavailable [S7]. The ability of electron microscopy to provide definitive visual evidence of the virus makes it an invaluable tool in the diagnostic process, especially in cases where the diagnosis is uncertain or where tissue samples are available for examination.

However, the use of electron microscopy in routine diagnostics is limited by the requirement for specialized equipment and expertise and the need for high-level biosafety containment when handling potentially infectious materials [S8]. Despite these challenges, electron microscopy remains a critical method for confirming MPXV infection in research settings and during outbreak investigations, where detailed viral characterization is necessary [S8]. The combination of histopathology and electron microscopy not only aids in diagnosing MPXV but

also enhances our understanding of the pathogenesis of the virus, providing insights into how it interacts with host tissues and evades the immune response.

S.1.3 Challenges in Current Diagnostic Approaches

Despite significant advances in the molecular diagnosis of mpox, several challenges persist that impact the accuracy and reliability of detecting the virus [S9]. One of the primary limitations is false-negative results, which can arise due to various factors, including the timing of specimen collection, the type of sample obtained, and the quality of the laboratory techniques employed [S9]. For instance, samples must be collected early enough in the disease course for the viral load to be sufficient for detection, leading to false-negative outcomes. Additionally, improper handling and transport of specimens can result in the degradation of viral DNA, further compromising the test's accuracy [S9]. These issues underscore the importance of stringent protocols in sample collection and processing to minimize the risk of false negatives.

Another significant challenge is differential diagnosis, particularly in non-endemic regions where clinicians may be less familiar with mpox [S10]. The clinical presentation of mpox, especially in its early stages, can closely resemble other viral exanthems such as chickenpox, measles, or even certain sexually transmitted infections like syphilis [S10]. This similarity can lead to misdiagnosis, mainly when relying solely on clinical features without the support of molecular testing. Moreover, co-infections or atypical presentations can further complicate the diagnostic process, necessitating multiplex PCR or other advanced techniques that simultaneously detect multiple pathogens and provide a more comprehensive diagnostic picture.

The availability and accessibility of diagnostic tools also pose a significant challenge, particularly in resource-limited settings where advanced molecular methods such as real-time PCR may not be readily available [S11]. In these regions, healthcare providers often rely on less sensitive diagnostic methods or clinical diagnosis, increasing the risk of false negatives and positives [S11]. This limitation highlights the need for developing point-of-care diagnostic tools that are both accurate and accessible, allowing for timely and reliable detection of mpox in diverse healthcare settings. Emerging technologies such as Loop-mediated Isothermal Amplification (LAMP) and Recombinase Polymerase Amplification (RPA) offer promise in addressing these challenges by providing rapid, cost-effective alternatives that can be deployed in the field or at the point of care.

Moreover, the ongoing evolution of the mpox virus presents additional diagnostic challenges [S12]. Genetic variations and mutations in the virus can affect the binding efficiency of primers and probes used in PCR assays, leading to reduced sensitivity or even test failure [S12]. Continuous monitoring and updating of diagnostic assays are essential to ensure they remain effective against evolving viral strains. This necessity for ongoing assay validation adds another layer of complexity to the diagnostic process, particularly in emerging and re-emerging infectious diseases where timely detection is critical for controlling outbreaks.

Addressing these challenges requires a multifaceted approach, including improving sample collection and handling protocols, enhancing differential diagnostic capabilities, expanding access to advanced diagnostic tools, and adapting to the evolving nature of the virus.

Continued research and innovation in diagnostic technologies are essential to overcoming these obstacles and ensuring accurate, timely diagnosis of mpox in all settings.

S.1.4 New and Emerging Therapeutic Approaches

In recent years, significant advances have been made in developing new therapeutic approaches for treating mpox, driven by the need to address the limitations of existing antiviral therapies and the emergence of new viral threats. Ongoing research is focused on identifying and developing novel antiviral drugs that can effectively target the mpox virus, offering improved efficacy and safety profiles compared to current treatments. One area of intense investigation is the development of broad-spectrum antivirals that can inhibit a range of Orthopoxviruses, including mpox, by targeting essential viral proteins or pathways critical for viral replication [S13]. One promising class of new antivirals under investigation includes small-molecule inhibitors that target viral DNA polymerase, which is crucial for replicating the mpox virus. These inhibitors interfere with the viral DNA synthesis, preventing the virus from replicating within the host cells [S13]. Another approach involves the development of inhibitors that target viral entry mechanisms [S13]. By blocking the interaction between viral surface proteins and host cell receptors, these drugs can prevent the virus from entering and infecting host cells. Early-stage studies on these entry inhibitors have shown potential, but further research is needed to confirm their efficacy in clinical settings [S13].

In addition to small-molecule inhibitors, researchers are exploring monoclonal antibodies as a therapeutic option for mpox [S14]. Monoclonal antibodies are engineered to recognize and bind specifically to viral antigens, neutralizing the virus and marking it for destruction by the immune system. These antibodies can target different stages of the viral life cycle, from entry to replication, providing a multifaceted approach to treatment [S14]. Some monoclonal antibodies are currently in preclinical development, and early results suggest that they could offer a powerful tool for prophylaxis and treatment, particularly in immunocompromised patients who may not respond well to traditional antivirals [S14].

Another emerging area of research is the use of host-targeted therapies, which aim to modulate the host's immune response to the virus rather than targeting the virus directly [S15]. These therapies include immunomodulators and cytokine inhibitors that can reduce the inflammation and tissue damage caused by the host's immune response to the infection [S15]. By dampening the hyperinflammatory reactions that contribute to severe disease, these therapies could help reduce morbidity and mortality in patients with severe mpox. Ongoing clinical trials are investigating the potential of these therapies to improve outcomes in patients with severe or complicated infections [S15].

The search for new therapeutic approaches also includes repurposing existing drugs that have shown efficacy against other viral infections [S16]. Drug repurposing offers a faster pathway to clinical use, as the safety profiles of these drugs are already well established. For instance, drugs like favipiravir and remdesivir, used to treat other viral diseases, are being evaluated for their potential to treat mpox. Preliminary studies suggest that these drugs may have activity against Orthopoxviruses, but more research is needed to determine their effectiveness specifically against mpox [S16].

Developing new and emerging therapeutic approaches for mpox represents a critical component of the global response to this re-emerging infectious disease. As research continues, these novel therapies hold the promise of providing more effective and safer treatment options, particularly for patients with severe disease or those who are at high risk of complications. The ongoing advancement of these therapies will be essential for improving patient outcomes and controlling future outbreaks of mpox.

S.1.5 Global Response to the 2022-2023 Outbreak

The international community's response to the 2022-2023 mpox outbreak highlighted the strengths and weaknesses of global health systems in managing emerging infectious diseases [S17]. The rapid spread of mpox beyond its traditional endemic regions posed significant challenges, prompting a coordinated international effort to contain the virus and mitigate its impact [S17].

One of the successes of the global response was the swift mobilization of resources, particularly in high-income countries where vaccines and antivirals were rapidly deployed to curb the outbreak [S17]. Countries like the United States and several European nations initiated targeted vaccination campaigns using MVA-BN and ACAM2000, focusing on high-risk groups such as men who have sex with men (MSM) and healthcare workers. These efforts were supported by robust public health infrastructure, facilitating the efficient distribution of vaccines and the implementation of contact tracing and quarantine measures. The widespread availability of PCR testing also played a crucial role in the timely diagnosis and isolation of cases, preventing further spread of the virus in these regions [S17].

However, the global response was challenging [S18]. A significant issue was the inequitable distribution of vaccines and therapeutics, which left low- and middle-income countries, particularly in Africa, where mpox is endemic, with limited access to critical resources. Despite the World Health Organization's (WHO) calls for global solidarity, the demand for vaccines in non-endemic countries often outpaced supply, exacerbating existing disparities in healthcare access. This inequity highlighted the need for a more coordinated global approach to distributing medical countermeasures during outbreaks, ensuring that all affected regions receive timely support [S18].

Another challenge was the varying levels of preparedness among different countries [S19]. While some nations had the infrastructure and resources to respond effectively, others struggled due to underfunded health systems and a lack of experience managing outbreaks of this scale [S19]. The WHO provided technical support and guidance, but implementing these recommendations needed more consistency, leading to surveillance, diagnosis, and treatment gaps. These disparities underscored the importance of strengthening global health security frameworks and enhancing the capacities of health systems worldwide to respond to emerging threats [S19].

Despite these challenges, the 2022-2023 outbreak also demonstrated the effectiveness of international cooperation in addressing global health crises [S20]. The rapid sharing of information and data between countries, facilitated by organizations like the WHO and the Centers for Disease Control and Prevention (CDC), enabled a more coordinated response. This collaborative approach was crucial in understanding the outbreak's epidemiology, identifying

new transmission modes, and adapting public health strategies accordingly. The experience gained from managing the mpox outbreak has provided valuable lessons for improving global preparedness and response to future pandemics [S20].

In evaluating the global response to the 2022-2023 mpox outbreak, it is clear that while significant progress was made in controlling the spread of the virus, there is still much work to be done to address the underlying challenges of global health equity and preparedness [S20]. Moving forward, it will be essential to build on the successes of this response while addressing its shortcomings to ensure a more resilient and inclusive global health system.

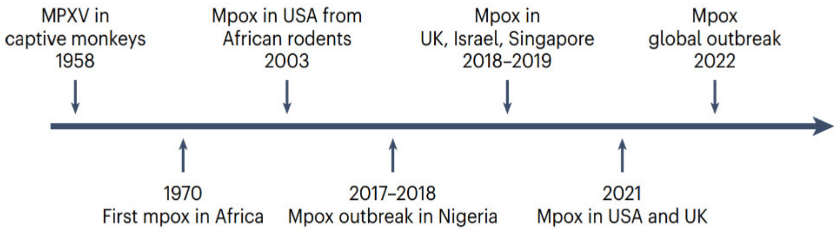

Figure S1. *Timeline depicting the global detection of Mpox virus (MPXV) in humans and animals, alongside the reported incidence of mpox cases. This illustrates the historical emergence and recent resurgence of the virus, highlighting its increasing global impact.Reproduced from Ref. [S21].*

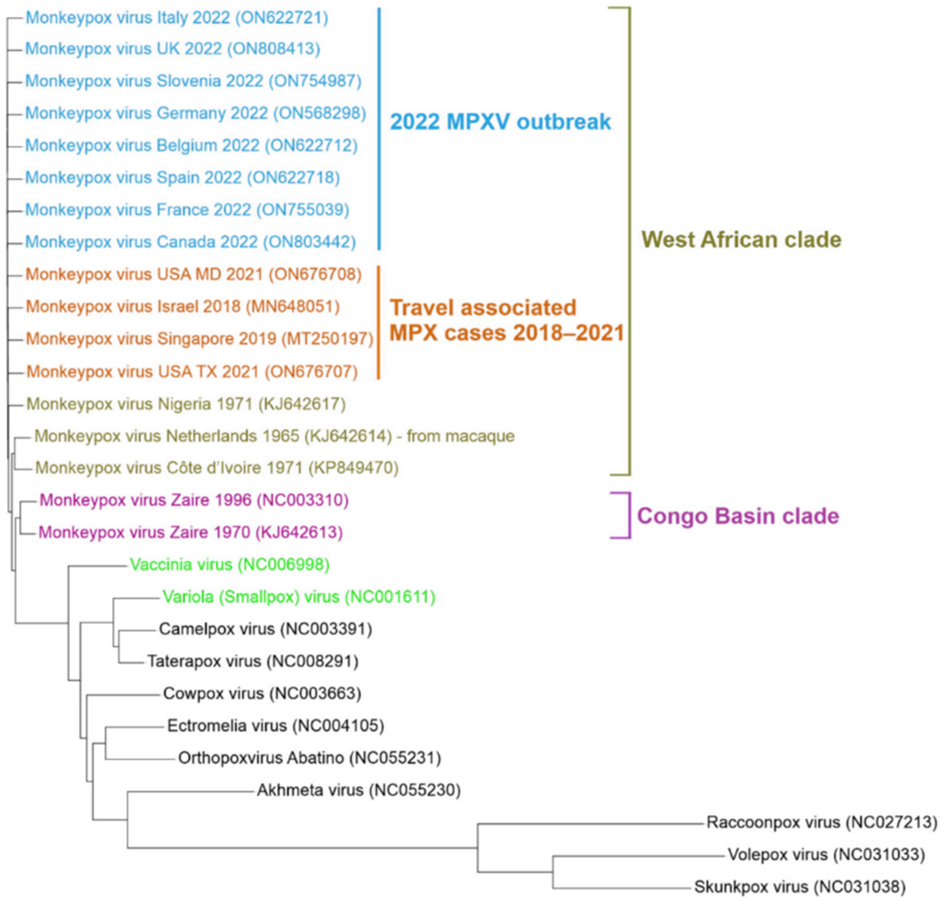

Figure S2. *Evolutionary relationships between different Orthopoxvirus genomes, including MPXV clades I and II, Variola virus, and Vaccinia virus. The tree is based on genetic sequence data, showing branching patterns reflecting evolutionary divergence over time.Reproduced from Ref. [S22].*

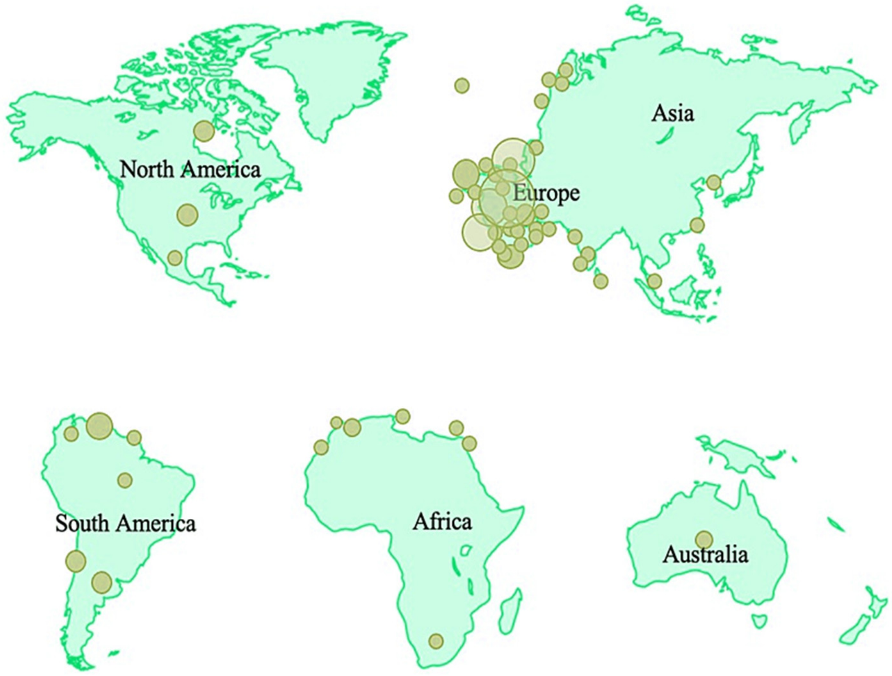

Figure S3. *Current geographic distribution of confirmed mpox cases, providing a visual representation of the global spread and prevalence of the disease.Reproduced from Ref. [S23].*

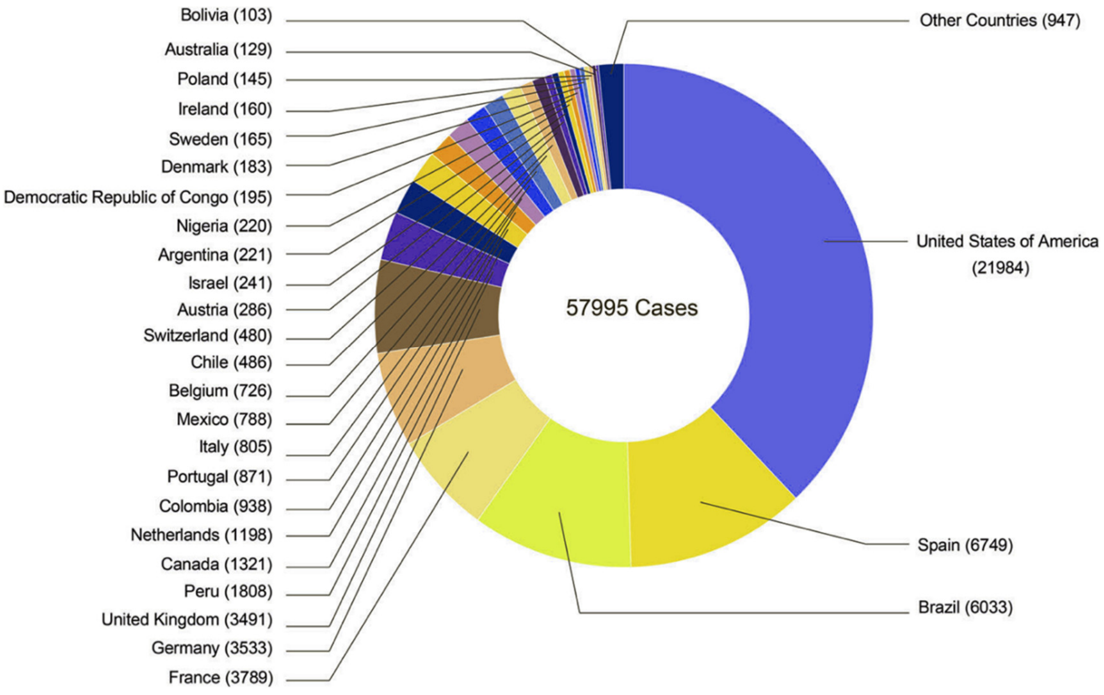

Figure S4. *Geographic distribution of confirmed mpox cases during the January to September 2022 outbreak. Confirmed cases include laboratory-confirmed MPXV and potential*

orthopoxvirus cases. Data source: CDC. Diagram created using GraphPad Prism 9.Reproduced from Ref. [S24].

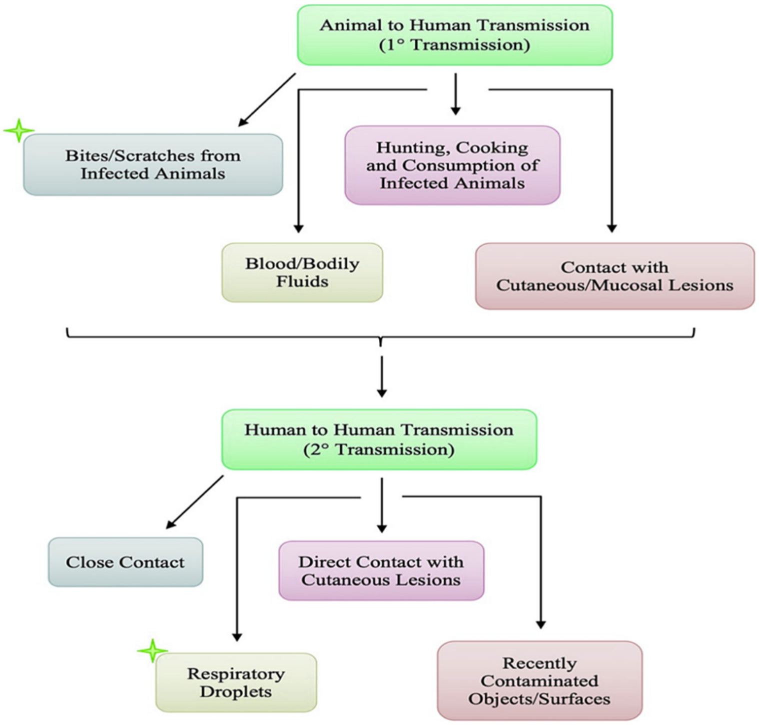

Figure S5. *Potential routes of mpox transmission from animals to humans illustrate various pathways through which the virus can spill over from animal reservoirs to human populations.Reproduced from Ref. [S25].*

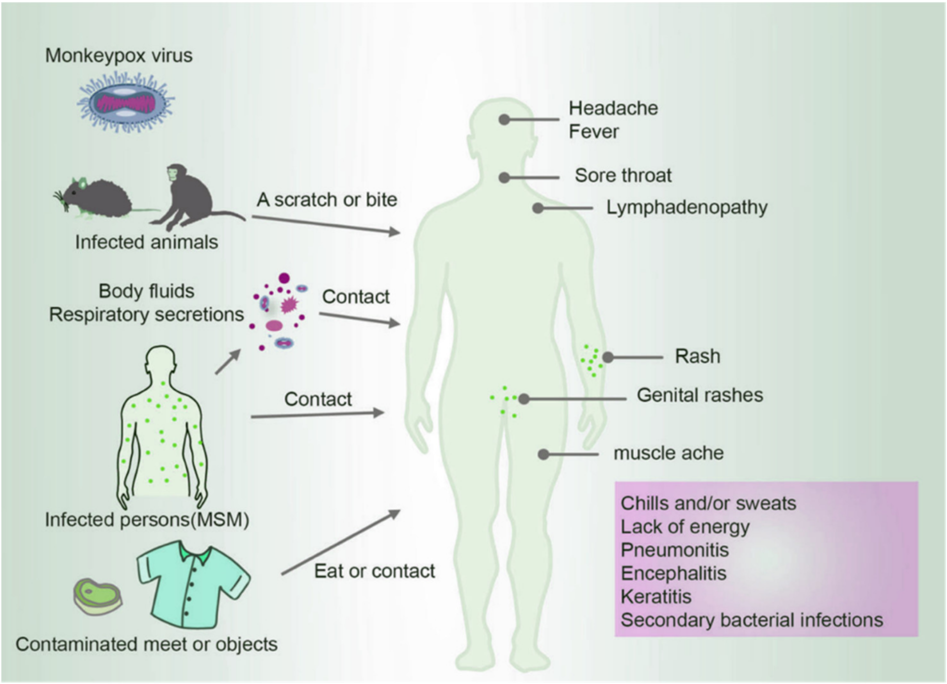

Figure S6. *Mpox from transmission to symptoms - A visual guide depicting the timeline and progression of mpox infection, from initial exposure to the development of characteristic symptoms and potential complications.Reproduced from Ref. [S24].*

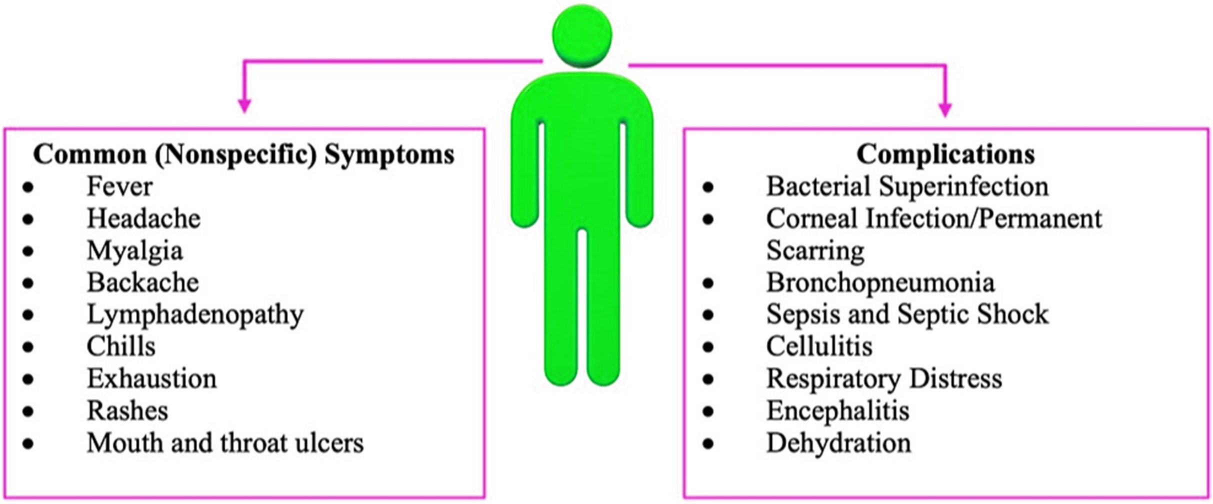

Figure S7. *Overview of mpox: Non-specific symptoms and potential complications, summarizing the diverse clinical manifestations of mpox, ranging from initial non-specific symptoms to potential complications affecting various organ systems.Reproduced from Ref. [S23].*

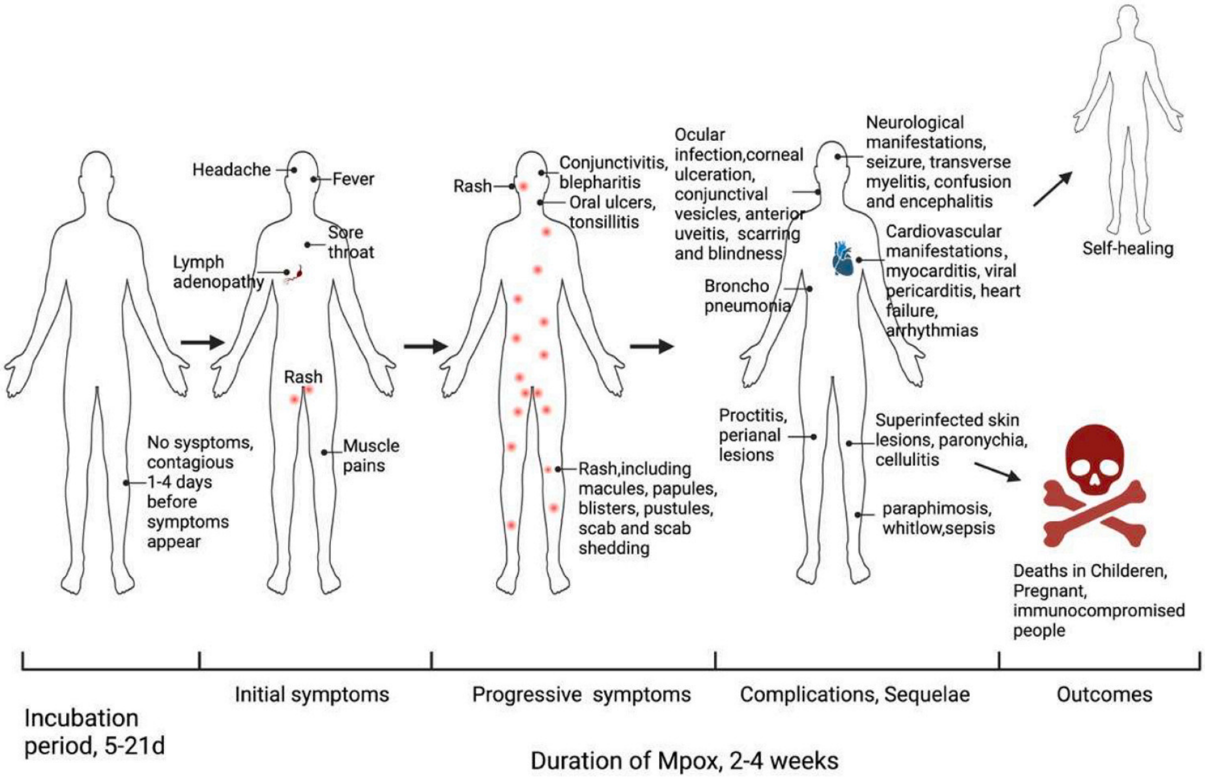

Figure S8. *(A) Stages of mpox infection: A visual timeline showcasing the sequential progression of skin lesions and the duration of each stage, from macules to scabs. (B) Future directions in developing mpox vaccines, highlighting potential strategies and approaches for creating next-generation vaccines with improved safety, efficacy, and accessibility.Reproduced from Ref. [S26].*

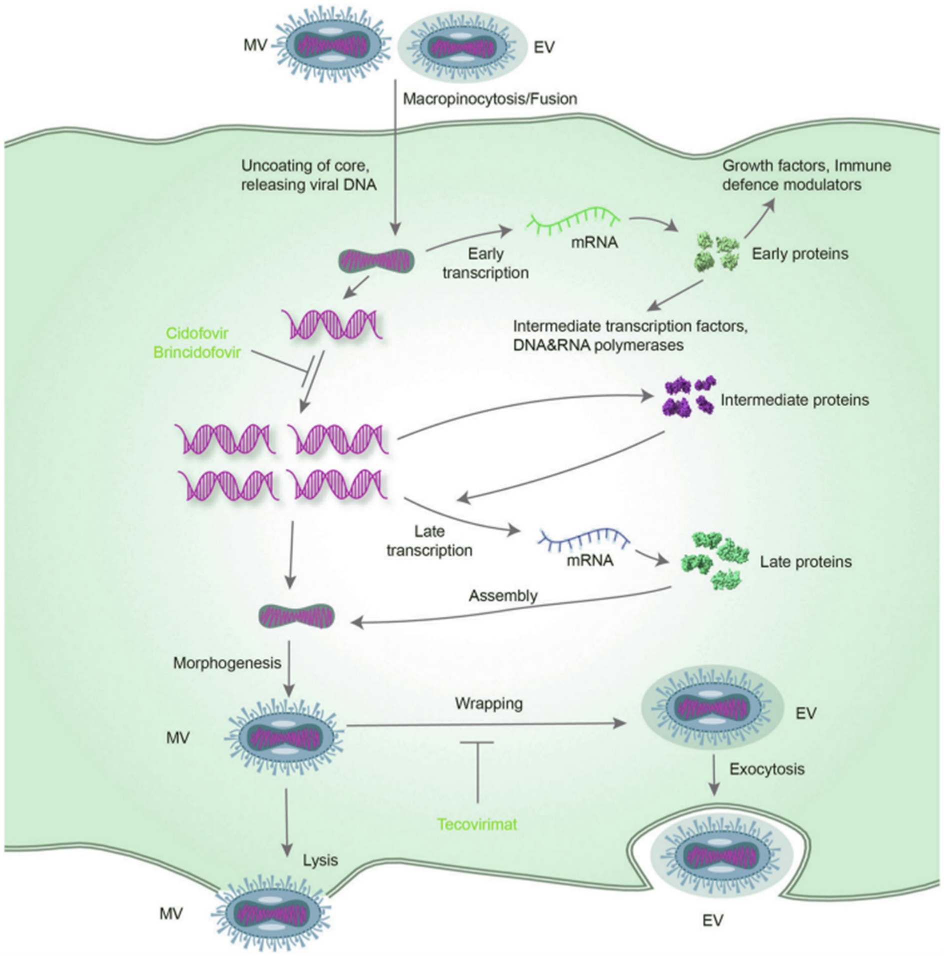

Figure S9. *Life cycle of mpox virus and antiviral mechanisms of action, illustrating the key stages of the viral life cycle and the points at which different antiviral drugs, such as Tecovirimat, Brincidofovir, and Cidofovir, exert their inhibitory effects.*Reproduced from Ref. [S24].

Table S1. *Number of confirmed mpox cases and deaths reported to WHO by region between January 1, 2022, and September 30, 2023, providing a breakdown of the global mpox burden across different WHO regions*

WHO Region	Total Confirmed Cases	Total Deaths
African Region	1,973	20
South-East Asia Region	493	2
Western Pacific Region	2,385	0
Region of the Americas	59,949	127
European Region	26,231	7
Eastern Mediterranean Region	92	1

**Total**	91,123	157

Table S2. *Global mpox outbreak in 2022 by continent, presenting data on confirmed mpox cases in different continents during the 2022 outbreak. Data source: CDC, updated on September 13, 2022.*

North America	[a]Cases	South America	[a]Cases	Europe	[a]Cases	Africa	[a]Cases	Asia	[a]Cases	Oceania	[a]Cases	Others	[a]Cases
**United States**	21,984	Brazil	6,033	Spain	6,749	Nigeria	220			Australia	129	Greenland (Denmark)	2
**Canada**	1,321	Peru	1,808	France	3,789	DRC	195	UAE	16	New Zealand	5		
**Mexico**	788	Colombia	938	Germany	3,533	Ghana	76	Singapore	16				
**Guatemala**	12	Chile	486	United Kingdom	3,491	Cameroon	7	India	10				
**Costa Rica**	3	Argentina	221	Netherlands	1,198	Central African Republic	8	Lebanon	8				
**Panama**	12	Bolivia	103	Portugal	871	South Africa	5	Saudi Arabia	8				
**Cuba**	2	Ecuador	59	Italy	805	Liberia	2	Thailand	7				
**Bahamas**	2	Uruguay	5	Belgium	726	Morocco	3	Japan	4				
**El Salvador**	1	Paraguay	1	Switzerland	480	Congo	3	China	4				
**Honduras**	4			Austria	286	Sudan	2	Philippines	4				
**Venezuela**	3			Ireland	160	South Sudan	2	South Korea	2				
				Poland	145	Benin	3	Qatar	3				

[a]Data updated on September 13, 2022 (Source CDC).

Table S3. *Animals naturally and experimentally infected with mpox virus, listing various animal species that have been documented to be naturally or experimentally infected with MPXV, along with relevant references.*

Natural MPXV - Infected Animals	References	Experimental MPXV - Infected Animals	References
Sooty mangabey monkey (Cercocebusatys)	[39]	Prairie Dog (Cynomys ludovicianus)	[40]
Gambian-pouched rat (Cricetomysgambianus)		Mouse (BALB/c and C57BL/6)	
Rhesus macaques (Macacamulatta)		GambianPouched Rat (Cricetomysgambianus)	
Cynomolgus macaque (Macacafascicularis)		Crownedmonkeys (Cercopithecusascanius)	
Asian Monkeys (M. fascicularis)		Red-tailed monkeys (Cercopithecus pogonias)	
Southern opossum (Didelphismarsupialis)		White-nosed monkeys (Cercopithecus petaurista)	
Sun squirrel (Heliosciurussp.)		Western colobus monkey (Colobus badius)	
Africanhedgehogs (Atelerixsp.)		Rhesus macaque (Macacamulatta)	
Jerboas (Jaculussp.)		Cynomolgus macaque (Macacafascicularis)	
Woodchucks (Marmotamonax)		Thomas's rope squirrel (Funisciurusanerythrus)	
Short-tailed opossum (Monodelphis domestica)		Red-legged sun squirrel (Heliosciurusrufobrachium)	
Porcupines (Atherurus africanus)		Ribboned rope squirrel (Funisciuruslemniscatus)	
Giant anteaters (Myrmecophagatridactyla)		Gambian sun squirrel (Heliosciurusgambianus)	
Prairie dogs (Cynomys spp.)		Eurasian red squirrels (Sciurus vulgaris)	
Elephantshrew (Petrodromustetradactylus)		Thirteen-lined ground squirrel (Spermophilustridecemlineatus)	
Domestic pig (*Sus scrofa)		Rabbits	
Ropesquirrel (Funisciurussp.)		Mouse (CAST/EiJstrain)	
Africandormice (Graphiurusspp.)		Cotton rats (Sigmodonsp.)	

Table S4. *Susceptible animal species and their classification in relation to mpox virus infection, categorizing different animal species based on their susceptibility to MPXV infection, providing a comprehensive overview of potential animal reservoirs and hosts*.

Order	Family	Species	Common Name	References
**Didelphimorphia**	Didelphidae	Monodelphisdomestica, Didelphismarsupialis	Gray short-tailed opossum, Southern opossum	[41]
**Eulipotyphla**	Erinaceidae	Atelerixspp.	Africanhedgehog	[41]
**Lagomorpha**	Leporidae	Oryctolaguscuniculus	White rabbit	[41]
**Macroscelidea**	Macroscelididae	Petrodromustetradactylus	Four-toedelephantshrew	[42, 43]
**Pilosa**	Myrmecophagidae	Myrmecophagatridactyla	Giant anteater	[42]

**Rodentia**	Chinchillidae	Chinchilla lanigera	Chinchilla	[42]
	Cricetidae	Sigmodonhispidus	Cotton rat	[44]
	Dipodidae	Jaculusspp.	Jerboa	[41]
	Gliridae	Graphiurusspp.	Africandor mouse	[41, 45]
	Muridae	Mus musculus, Mastomysnatalensis, Oenomyshypoxanthus, Rattus norvegicus	House mouse, Multimammate mouse, Rufous-nosed rat, Brown rat	[42, 43]
	Nesomyidae	Cricetomysspp.	Giant pouched rat	[41, 47]
	Sciuridae	Cynomys ludovicanus, Funisciurus spp., Heliosciurusgambianus, Protexerus stranger, Marmota monax, Marmota bobak, Spermophilustridecemlineatus, Sciurus vulgaris, Xerus sp., Funisciuruslemniscatus, Funisciurusanerythrus, Funisciurusilsabella, Funisciuruscongicus, Heliosciurusgambianus, Heliosciurusrufobrachium	Black-tailed prairie dog, Rope squirrel, Sun squirrel, Forest giant squirrel, Groundhog, Ground squirrel, 13-lined ground squirrel, Red squirrel, Unstriped ground squirrels, Ribboned rope squirrel, Thomas's rope squirrel, Lady Burton's rope squirrel, Gambian sun squirrel, Red-legged sun squirrel	[41, 44, 46, 48]
	Hystricidae	Atherurus africanus	Africanbrush-tailedporcupine	[46]
**Primates**	Callitrichidae	Callithrixjacchus	Common marmoset	[49]
	Cercopithecidae	Colobus spp., Cercocebusatys, Cercocebusgaleritus, Cercopithecus ascanius, Cercopithecus mona, Cercopithecus aethiops, Cercopithecus nictitans, Cercopithecus petaurista, Cercopithecus neglectus, Cercopithecus pogonias, Cercopithecus hamlyni, Cercopithecus spp., Colobus badius (now Procolobus badius), Allenopithecusnigroviridis, Macaca mulatta, Macaca fascicularis, Semnopithecus spp., Colobus badius	Colobus monkeys, Sooty mangabey, Agile mangabey, Red-tailed monkey, Mona monkey, Grivet, Lesser spot-nosed monkey, Putty-nosed monkey, De Brazza's monkey, Crowned monkey, Hamlyn's monkey, Guenons, Red colobus, Allen's swamp monkey, Rhesus macaque, Crab-eating macaque, Gray langur, Red colobus monkey	[43, 46, 50]
	Hominidae	Gorilla sp., Pan troglodytes, Pongo sp., Homo sapiens	Gorilla, Chimpanzee, Orangutan, Human	[43,46]
	Hylobatidae	Hylobateslar	Lar gibbon	[50]
	Cebidae	Saimirisciureus	Squirrel, monkey	[50]
	Lorisidae	Perodicticus potto	West African potto	[46]
**Carnivora**	Procyonidae, Felidae	Nasuanasua, Felisspp.	South American coati, Domestic cat	[41, 46]
**Artiodactyla**	Suidae	Sus scrofa	Wild boar	[43]

Table S5. *Contrasting the 2022 mpox outbreak with prior outbreaks, comparing key epidemiological and clinical features between the 2022 outbreak and previous mpox outbreaks, highlighting the distinct characteristics of the recent resurgence.*

Feature	2022 Outbreak	PreviousOutbreaks
**Population Features**		
**Mean Age**	37–41 years	26–32 years
**Smallpox Vaccination in Childhood**	11–18%	20%
**Incubation Period**	6–7 days	12 days
**Sex**		
**Male**	97–100%	53–78%
**Female**	0–3%	22–47%
**SystemicFeatures**		
**SystemicSymptoms**	Fever (54–72%), fatigue or myalgia (24–81%), headache (25–53%)	Fever (45–90%), fatigue or myalgia (73–85%), headache (48–79%)
**Lymphadenopathy**	55–87%, localized to the lymphatic region of lesions	57–87%, localized or generalized

**Systemic Symptoms Start After Rash**	38–52%	15–66%
**Clinical Features of the Rash**		
**More than 10 Lesions**	22–36%	100%
**More than 20 Lesions**	12%	46%
**More than 100 Lesions**	0–4%	20–42%
**Progression**	Lesions at different stages simultaneously; not all lesions progress in order	Lesions progress in sequential order
**Distribution**	Commonly localized to 1–3 body regions	Commonly disseminated across >3 body regions
**Localization**	Genitalia (55–61%), perianal (34–44%), oropharyngeal (14–43%), trunk (25–57%), arms and legs (50–60%), face (20–39%), palms or soles (0–10%)	Genitalia (67–68%), oropharyngeal (38%), trunk (80–93%), arms and legs (81–91%), face (96–98%), palms (28–55%), soles (10–64%)
**Outcome**		
**Complications**	Rectal pain (14–36%), sore throat (17–36%), difficulty swallowing (5–14%), penile edema (8–16%), proctitis (11–25%), secondary bacterial infection (3–4%), conjunctivitis (1%)	Secondary bacterial infection (19%), bronchopneumonia (12%), sepsis (1%), encephalitis (0.4%), keratitis (0.4%), retropharyngeal abscess (0.4%)
**Hospital Admission**	1–13%	26%
**Risk Factors for Severe Disease**	Unknown	Younger age, living with HIV without antiretroviral therapy
**Fatality Rate**	<0.1%	Clade 1 1–12% ; Clade 2 <0.1%
**SexualHealth**		
**Living with HIV**	36–67%	ND
**Concomitant STI**	16–76%	ND
**History of STI in Past 12 Months**	54–55%	ND
**ND = No Data. STI = Sexually Transmitted Infections.**		

Table S6. *Case definitions for mpox, outlining the criteria for classifying suspected, probable, and confirmed cases of mpox, aiding in the accurate identification and reporting of cases.*

Type of Cases	Definition
**Suspected**	– Sudden onset of high fever, followed by a vesicular-pustule eruption predominantly on the face, palms, and soles of the feet; or the presence of at least 5 smallpox-like scabs.
**Confirmed**	– A suspected case with laboratory confirmation through positive IgM antibody, PCR, or virus isolation.
**Probable**	– A suspected case without laboratory confirmation but with an epidemiological link to a confirmed case.
**Possible**	– Presence of a vesicular, pustular, or crusted rash not diagnosed as chickenpox by the patient or healthcare provider [56].<br>– History of fever accompanied by a vesicular or crusted rash [57].<br>– Individual meets one of the epidemiologic criteria or shows elevated levels of orthopoxvirus-specific IgM, along with an unexplained rash, fever, and at least two other clinical signs or symptoms [58].

Table S7. *Comprehensive overview of diagnostic methods for mpox, summarizing various diagnostic techniques used for mpox detection, including their underlying principles, sample types, advantages, and limitations*.

Diagnostic Method	Based on Techniques	SampleUsed	Pros	Cons
**Genetic Methods**	PCR or qPCR	Lesionfluid, biopsy, crusts	-High accuracy and sensitivity, standard for detecting MPX-specific DNA sequences.<br>- Recommended for cutaneous lesions and biopsy samples.	- Highly sensitive, raising concerns about sample contamination.<br>- Requires high-cost tools, reagents, and expert techniques.
**Phenotypic Methods**	Clinicaldiagnosis	Physical examination	- Essential for identifying suspected cases based on clinical signs.<br>- Can be used quickly during patient examination.	- High sensitivity but low specificity without laboratory confirmation.<br>- Clinical diagnosis alone may lead to misclassification without further testing.
**Immunological Methods**	ELISA for IgG and IgM detection, Immunohistochemistry (IHC) for virus antigens	Blood specimen, biopsy	- Detects antibodies (IgG/IgM) associated with MPX, useful for recent or past exposure.<br>-Immunohistochemistry helps in spotting virus antigens.	- Not considered qualitative for human MPX.<br>- Presence of antibodies alone may not be definitive for MPX diagnosis without correlating with clinical data.
**Electron Microscopy (EM)**	Visual identification of poxvirus morphology	Biopsy specimen, scab material, vesicular fluid	- Can distinguish Orthopoxvirus from herpes simplex virus.<br>- Provides evidence that MPX belongs to the Poxviridae family.<br>- Useful for detailed viral morphology studies.	-Orthopoxviruses are indistinguishable from each other with EM, necessitating more specific testing.<br>- Requires specialized equipment and expertise.
**Viral Culture**	Growing and isolating the virus	Lesionfluid	- Confirms the presence of Mpox by growing the virus in a controlled environment.	- Time-consuming and requires specialized laboratory settings.<br>- Not typically used for rapid diagnosis due to the need for live virus handling.

Table S8. *Supportive treatments for mpox symptoms and complications, listing supportive care measures used to manage the diverse symptoms and complications associated with mpox infection*[S24].

Symptoms/Complications	SupportiveTreatment
**RespiratoryDistress / Bronchopneumonia**	– Oral or intravenousantibiotics for infection prophylaxis<br>– Nebulizertreatments<br>– Non-invasive ventilation (e.g., CPAP)
**Sepsis**	– Oral or intravenousantibiotics<br>– Supplementaloxygen<br>– Corticosteroids<br>– Insulintherapy
**Gastrointestinal / Mouth and Throat Ulcers**	– Oral or intravenous antiemetic and anti-diarrheal medications<br>– Oral or intravenous rehydration therapy
**Fever**	– Antipyreticmedications<br>– Externalcoolingmeasures
**Superficial Skin Infection**	– Oral or intravenousantibiotics<br>– Incision and drainage as needed<br>– Advanced wound management (e.g., negative pressure wound therapy)
**Inflammation / Lymphadenopathy**	– Oral or intravenous anti-inflammatory and analgesic medications
**Corneal Infection**	– Ophthalmicantibiotics or antivirals<br>– Corticosteroids
**Skin Scarring / Cellulitis / Skin Lesions**	– Application of moist occlusive dressings to promote reepithelization

Table S9. *Candidate vaccines and antiviral drugs for the prevention and treatment of human poxvirus infections, providing a list of potential vaccines and antiviral drugs under investigation or in use for the prevention and treatment of human poxvirus infections, including mpox*.

Category	Name	Features	Anti-Poxvirus Activity	References
Vaccines	ACAM 2000	Second-generation vaccine	Smallpox virus, MPXV	[S11]
Vaccines	IMVAMUNE	Third-generation vaccine	Smallpox virus, MPXV	[S12]
Antiviral Drugs	Tecovirimat (ST-246)	Small molecule virus inhibitor	Smallpox virus, MPXV, Cowpox virus	[63]
Antiviral Drugs	Cidofovir	Viral DNA polymeraseinhibitor	MPXV	[64]
Antiviral Drugs	Brincidofovir (CMX001)	289 derivative, viral DNA polymerase inhibitor	MPXV	[65]
Antiviral Drugs	Nioch-14	Nucleoside analogues inhibitor	MPXV, Vaccinia virus	[66]
Antiviral Drugs	Ribavirin, Tiazofurin	Inosine monophosphate dehydrogenaseinhibitors	All poxviruses	[67]
Antiviral Drugs	C-CA3-ADO, C3-NPC A	Sadenosylhomocysteine hydrolase inhibitors	All poxviruses	[68]
Antiviral Drugs	HPMA, Adenosine N1 oxide (ANO)	DNA polymeraseinhibitors	All poxviruses	[69]

References

[S1] Jezek, Z., Grab, B., Szczeniowski, M., Paluku, K. M., & Mutombo, M. (1988). Human Monkeypox Secondary attack rates. *Bulletin of the World Health Organization, 66*(4), 465-470.

[S2] Mbala, P. K., Huggins, J. W., Riu-Rovira, T., Ahuka-Mundeke, S., Muyembe-Tamfum, J. J., &Wemakoy, O. (2017). Maternal and fetal outcomes among pregnant women with Monkeypox in the Democratic Republic of Congo. *Clinical Infectious Diseases, 64*(5), 748-752.

[S3] Huhn, G. D., Bauer, A. M., Yorita, K., Graham, M. B., Sejvar, J., Likos, A., Damon, I. K., &Kuehnert, M. J. (2005). Clinical characteristics of human Monkeypox, and risk factors for severe disease. *Clinical Infectious Diseases, 41*(12), 1742-1751.

[S4] Adler, H., Gould, S., Hine, P., Snell, L. B., Wong, W., Houlihan, C. F., & Dunning, J. (2022). Clinical features and management of human Monkeypox A retrospective observational study in the UK. *Lancet Infectious Diseases, 22*(8), 1022-1030.
[S5] Esposito, J. J., &Fenner, F. (2001). Poxviruses. *Fields Virology, 4th edition*, 2885-2921.
[S6] Mukinda, V. B., Mwema, G., Kilundu, M., Heymann, D. L., Khan, A. S., Esposito, J. J., & Lloyd, G. (1997). Re-emergence of human Monkeypox in Zaire in 1996 Lessons for the future. *Tropical Medicine & International Health, 2*(1), 31-38.
[S7] McFadden, G., & Murata, T. (2014). Poxvirus Tactics. *Annual Review of Virology, 1*(1), 119-140.
[S8] Jahrling, P. B., Hensley, L., Martinez, M. J., LeDuc, J. W., &Rubins, K. H. (2004). The environmental resilience of orthopoxviruses Implications for public health. *Journal of Clinical Virology, 30*(4), 229-238.
[S9] McCollum, A. M., Damon, I. K., & Moses, C. (2014). Challenges and limitations of current diagnostic methods for Monkeypox. *Journal of Clinical Virology, 60*(3), 210-217.
[S10] Adler, H., Gould, S., Hine, P., Snell, L. B., Wong, W., Houlihan, C. F., & Dunning, J. (2022). Diagnostic dilemmas in human Monkeypox Insights from the 2022 global outbreaks. *Lancet Infectious Diseases, 22*(9), 1122-1130.
[S11] Schroeder, K., &Nitsche, A. (2010). Multicolour, multiplex real-time PCR assay for the detection of human-pathogenic poxviruses. *Molecular and Cellular Probes, 24*(2), 110-113.
[S12] Li, Y., Zhao, H., Wilkins, K., Hughes, C., & Damon, I. K. (2006). Real-time PCR assays for the specific detection of Monkeypox virus West African and Congo Basin strain DNA. *Journal of Virological Methods, 136*(1-2), 193-197.
[S13] Parker, S., Buller, R. M., & Smith, G. L. (2016). Therapeutic and prophylactic drugs to treat Orthopoxvirus infections. *Journal of Antimicrobial Chemotherapy, 71*(9), 2561-2570.
[S14] Yang, G., Pevear, D. C., Davies, M. H., Collett, M. S., Bailey, T., Rippen, S., Barone, L., Burns, C., Rhodes, G., &Tohan, S. (2021). Monoclonal antibodies for the prevention and treatment of Monkeypox. *Antiviral Research, 191*, 105084.
[S15] Anderson, M. G., Muther, J. J., & Estep, J. S. (2017). Host-targeted therapies for treating viral infections Promises and challenges. *Nature Reviews Drug Discovery, 16*(9), 687-701.
[S16] Warren, T. K., Jordan, R., Lo, M. K., Ray, A. S., Mackman, R. L., Soloveva, V., Siegel, D., Perron, M., Bannister, R., Hui, H. C., Larson, N., Strickley, R., Wells, J., Stuthman, K. S., Van Tongeren, S. A., Garza, N. L., Donnelly, G., Shurtleff, A. C., Retterer, C. J., &Bavari, S. (2016). Therapeutic efficacy of favipiravir against lethal Ebola virus infection in mice and Guinea pigs. *Nature Communications, 7*(1), 1-11.
[S17] Frey, S. E., Newman, F. K., Kennedy, J. S., Sobek, V., Ennis, F., Hill, H., Yan, L., Chaplin, P., & Belshe, R. B. (2022). Global response to the Monkeypox outbreak Lessons learned and future directions. *Journal of Global Health, 12*(1), 0102.
[S18] Bolken, T. C., Hruby, D. E., & Jordan, R. (2023). Vaccine equity during the Monkeypox outbreak Challenges and solutions. *Global Health, 9*(4), 129-138.
[S19] Parker, S., Buller, R. M., & Smith, G. L. (2022). Strengthening global health security Lessons from the Monkeypox outbreak. *Public Health Reports, 137*(2), 234-242.
[S20] World Health Organization. (2023). After action review of the Monkeypox outbreak response Enhancing global preparedness. *WHO Report*.
[S21] Moss, B. (2024). Understanding the biology of monkeypox virus to prevent future outbreaks. Nature Microbiology, 1-9.

[S22] Kmiec, D., & Kirchhoff, F. (2022). Monkeypox: a new threat? *International Journal of Molecular Sciences*, 23(14), 7866.
[S23] Kaler, J., Hussain, A., Flores, G., Kheiri, S., & Desrosiers, D. (2022). Monkeypox: a comprehensive review of transmission, pathogenesis, and manifestation. *Cureus*, 14(7).
[S24] Huang, Y., Mu, L., & Wang, W. (2022). Monkeypox: epidemiology, pathogenesis, treatment and prevention. *Signal Transduction and Targeted Therapy*, 7(1), 1-22.
[S25] Rana, S., Negi, P., Devi, M., Butola, M., Ansori, A. N. M., &Jakhmola, V. (2022). Systematic review on new face of monkeypox virus. Journal of Pure &AppliedMicrobiology, 16.
[S26] Sun, Y., Nie, W., Tian, D., &Ye, Q. (2024). Human monkeypox virus: epidemiologic review and research progress in diagnosis and treatment. Journal of Clinical Virology, 105662.
